# Climate pacing of millennial sea-level change variability in the central and western Mediterranean

**DOI:** 10.1038/s41467-021-24250-1

**Published:** 2021-06-29

**Authors:** Matteo Vacchi, Kristen M. Joyse, Robert E. Kopp, Nick Marriner, David Kaniewski, Alessio Rovere

**Affiliations:** 1 Dipartimento di Scienze Della Terra, Università di Pisa, Pisa, Italy; 2 CIRSEC—Centro Interdipartimentale di Ricerca per lo Studio degli Effetti del Cambiamento climatico dell’Università di Pisa, Pisa, Italy; 3 Department of Earth & Planetary Sciences and Rutgers Institute of Earth, Ocean, and Atmospheric Sciences, Rutgers University, New Brunswick, NJ USA; 4 CNRS, ThéMA, Université de Franche-Comté, UMR 6049, MSHE Ledoux, Besançon, Cedex France; 5 TRACES, UMR 5608 CNRS, Université Toulouse Jean Jaurès, Maison de la Recherche, Toulouse, Cedex 9 France; 6 MARUM, Center for Marine Environmental Sciences, University of Bremen, Bremen, Germany

**Keywords:** Palaeoceanography, Palaeoclimate, Physical oceanography

## Abstract

Future warming in the Mediterranean is expected to significantly exceed global values with unpredictable implications on the sea-level rise rates in the coming decades. Here, we apply an empirical-Bayesian spatio-temporal statistical model to a dataset of 401 sea-level index points from the central and western Mediterranean and reconstruct rates of sea-level change for the past 10,000 years. We demonstrate that the mean rates of Mediterranean industrial-era sea-level rise have been significantly faster than any other period since ~4000 years ago. We further highlight a previously unrecognized variability in Mediterranean sea-level change rates. In the Common Era, this variability correlates with the occurrence of major regional-scale cooling/warming episodes. Our data show a sea-level stabilization during the Late Antique Little Ice Age cold event, which interrupted a general rising trend of ~0.45 mm a^−1^ that characterized the warming episodes of the Common Era. By contrast, the Little Ice Age cold event had only minor regional effects on Mediterranean sea-level change rates.

## Abstract (web summary)

How sea-level in the western Mediterranean reacts to climate changes is not well known. Here, the authors present a regional reconstruction and show that temperatures influenced sea-level change rates during the Holocene, while recent sea-level rise is happening faster than during any other period of the last 4000 years.

## Introduction

Climate and sea-level reconstructions for the pre-industrial period (i.e., before 1850 CE) provide context for current and future changes^1–3^. Determining the rates, mechanisms, and geographic variability of relative sea-level (RSL) change following the Last Glacial Maximum (LGM) is relevant to gauging how climatic forcing may influence the rates of future sea-level change^3,4^. Compilations of sea-level proxies have facilitated the quantification of the response of the solid Earth and geoid to ice-mass redistribution^5–7^ and provided constraints for statistical and geophysical models used to project future sea-level rise^8^. The Mediterranean Sea is a semi-enclosed basin lying in a transition zone between mid-latitude and subtropical atmospheric circulation regimes and is characterized by strong land-sea contrasts^9^. For this reason, it is considered a hotspot of current climate change^9–11^, and future warming in the Mediterranean area is expected to exceed global rates by ∼25%^12^. This may result in high sea-level rise rates compared to global averages, leading to significant losses in the environmental, cultural and economic values of Mediterranean coasts^13^. Furthermore, semi-closed basins are poorly resolved by the ~1° resolution typical of global climate models included in CMIP5/6, which are generally used to drive projections of local dynamic sea-level change^14^. Finally, offset between data and glacio-isostatic adjustment (GIA) models were recently highlighted by extended sea-level records^15^. This adds complexity to defining the magnitude and spatial variability of the isostatic component, which affects both current and future sea-level changes.

The increasing availability of continuous and high-resolution Holocene and Common Era Mediterranean relative sea-level (RSL) reconstructions^15,16^ provides the opportunity to explore the role of climatic factors in mediating sea-level variability in the Holocene (i.e., the last 11.7 ka). These data are of major importance because regional climatic forcing can lead to significant departures from global mean sea-level projections^10^.

**Fig. 1:**
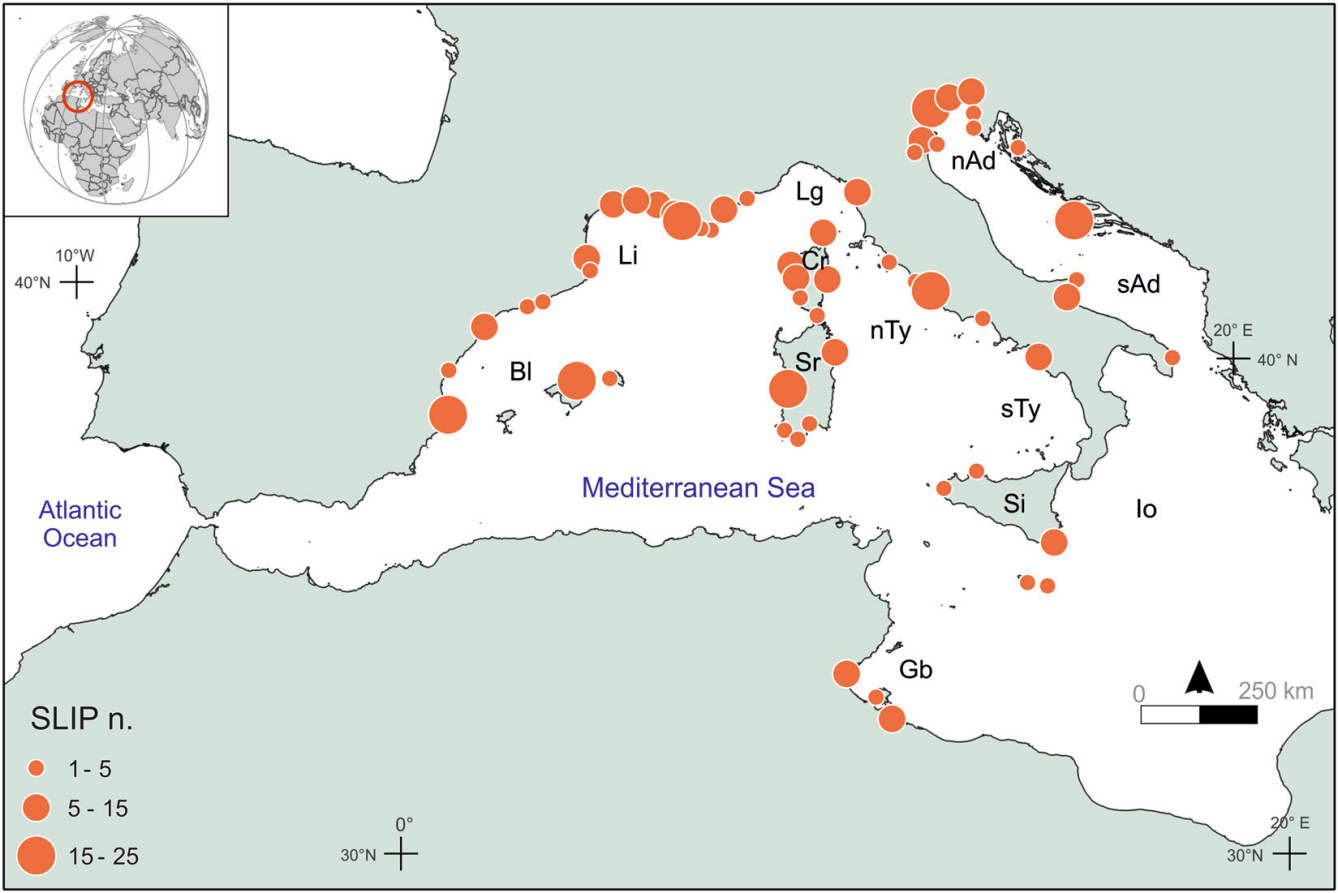
Spatial distribution of the central and western Mediterranean sea-level index points (SLIPs) used for this analysis. Br is the Balearic Sea; Li is the Gulf of Lion; Lg is the Ligurian Sea; nTy is the northern Tyrrhenian Sea; sTy is the southern Thyrrenian Sea; Gb is the Gulf of Gabes; Io is the Ionian Sea; nAd is the northern Adriatic; sAd is the southern Adriatic Sea; Sr is Sardinia Island; Cr is Corsica Island; Si is Sicily Island.

Here, we applied an empirical-Bayesian spatio-temporal statistical model^17^ (see “Methods”) to a dataset of 401 sea-level index points (SLIPs), defining the discrete position of past RSL in space and time^18^. We focus our analysis on the central and western Mediterranean (Fig. 1), which are less affected by neotectonic processes^19^ than the eastern part. The results of our analysis constitute the first basin-scale assessment of sea-level variability in the Mediterranean for the last 10,000 years and represent the natural and geological backgrounds against which modern Mediterranean sea-level rise should be quantified.

## Results and discussion

### Millennial variability of sea-level change rates

The SLIPs database (Supplementary Table 1) is composed of (i) 391 radiocarbon-dated geological samples from transitional brackish environments, fossil intertidal bioconstructions, beachrocks, and (ii) 12 archeologically dated marine structures whose relationship with the contemporary mean sea-level is robustly defined^20^. The spatial distribution of the SLIPs covers a large portion of the central and northern sectors of the central and western Mediterranean basin, while in the southern sector the available data are restricted to the coasts of Malta and the Gulf of Gabes (Fig. 1). The SLIPs database allowed us to reconstruct the rates of RSL change in 48 sub-regions clustered according to their geographic proximity (Supplementary Fig. 1a). The average vertical accuracy of the different SLIPs is ±0.7 m (max ±1.3 m, min ±0.2 m) while the average age error is ±0.15 ka (max 0.62 ka, min 0.025 ka). All errors are reported at ±1σ. The age of the SLIPs spans the whole Holocene period (Supplementary Fig. 1b). 10.3% of the SLIPs date to the early Holocene (−10,000 to −6000 CE), 32.4% to the mid-Holocene (−6000 to −2000 CE), and 57.3% to the late Holocene (−2000 to 1950 CE). Virtually uncompressible samples (see “Methods”) represent 36.5% of the entire record, while 39.4% of the SLIPs dates between −5000 and 1950 CE (Supplementary Fig. 1b).

**Fig. 2:**
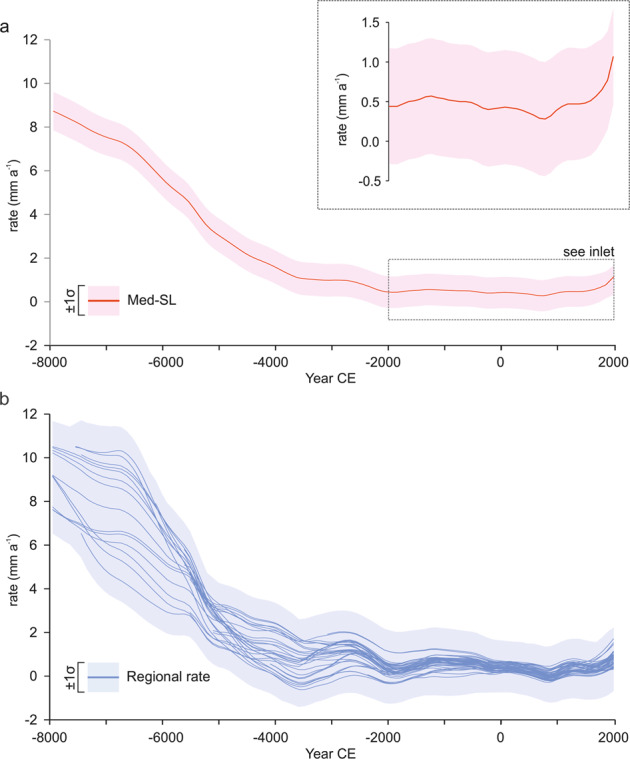
Rates of relative sea-level change for the central and western Mediterranean region in the last 10,000 years. **a** Common sea-level signal (Med-SL). The inlet graph shows the Med-SL variation in the last 4000 years. The solid line and shaded envelope denote the model mean and the 1σ uncertainty (see Supplementary Table 2). **b** variability of relative sea-level (RSL) change rates in the 48 central and western Mediterranean regions included in the analysis. The solid line and shaded envelope are the model mean and 1 s uncertainty.

The spatio-temporal model allowed us to reconstruct sea-level change rates since −8000 CE. There is a paucity of SLIPs for the period −10,000 to −8000 CE. From the model, it was possible to calculate the “central and western Mediterranean Sea-Level” (Med-SL), which represents the common signal found at all sites included in the model runs (Fig. 2a and Supplementary Table 2). The Med-SL, which is uniform over the entire central and western Mediterranean, absorbs a majority of the sea-level signal, whereas the regional signals (Fig. 2b, Supplementary Fig. 2) explain the variability we can observe at the basin scale. The model also estimates RSL for locations and times where there are no direct observations because it recognizes that an observation associated with a single point in space and time is informative about sea-level at proximal locations and times.

Med-SL estimates (Fig. 2a) indicate that the central and western Mediterranean sea-level rise decreased from 8.7 ± 0.9 mm a^−1^ to 3.1 ± 0.8 mm a^−1^ during the period −8000 to −5000 CE. The slowdown continued over subsequent millennia, with average rates of 1.5 ± 0.8 mm a^−1^ between −5000 and −2000 CE, 0.5 ±  0.7 mm a^−1^ between −2000 and 0 CE, and 0.45 ± 0.7 mm a^−1^ in the last 2000 years. This stabilization trend reflects the general decrease in rates of global sea-level change consistent with the final phase of North American deglaciation and the consequent sudden reduction of meltwater input and with the stabilization of global mean surface temperature^7,21^. Between −2000 and 1850 CE, the ice-equivalent meltwater input is either zero or minimal^21,22^. During this period, Med-SL rise rates ranged between 0.30 ± 0.7 mm a^−1^ and 0.55 ± 0.6 mm a^−1^, while in the industrial-era (e.g., post 1850 CE) rates increased up to 1.05 ± 0.6 mm a^−1^ (Fig. 2a, Supplementary Table 2). This acceleration closely mirrors post-industrial warming observed in several Mediterranean climatic proxies^12^ and is consistent with the data extracted from the longest central and western Mediterranean tide-gauge data, which indicate sea-level rise rates of about 1.2–1.3 mm a^−1 ^^23,24^ for the period 1880–2012 CE. Even higher rates (1.7–1.8 mm a^−1^) are observed for the second part of the last century, indicated by a larger dataset of tidal gauges and satellite altimetry^25,26^. This indicates that, at the basin scale, the mean estimate of industrial-era sea-level rising rate has no equivalent analog during the last 4000 years and that the rate of central and western Mediterranean industrial-era sea-level rise is unlikely (~25% probability) to be a random occurrence (Supplementary Fig. 3).

**Fig. 3:**
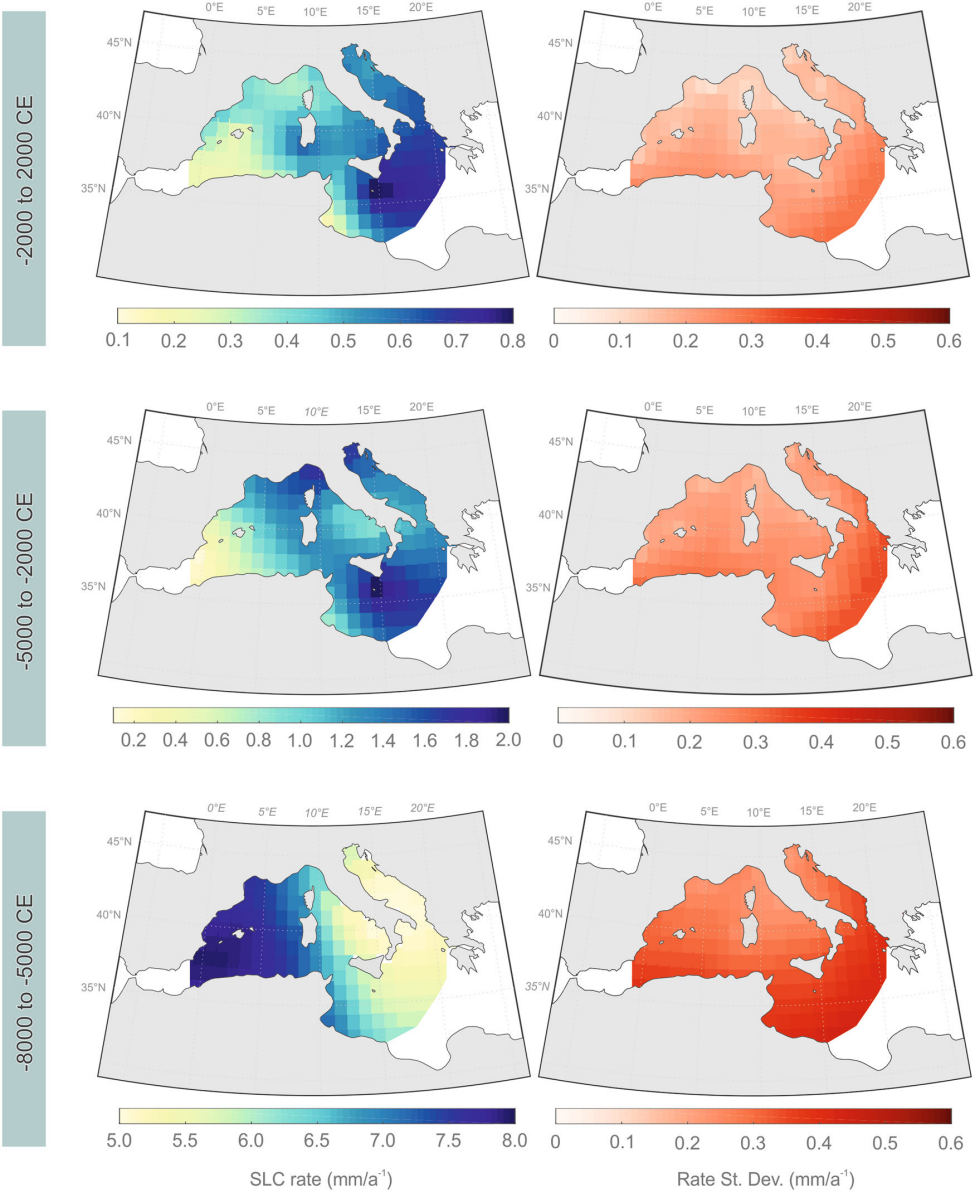
Spatial and temporal variability of relative sea-level (RSL) changes and their uncertainties across the central and western Mediterranean basin in the time periods 2000 to −2000 CE, −2000 to −5000 CE, and −5000 to −8000 CE. Note the changes of scale for the different time intervals.

Our analysis highlights significant variability in the regional rates of sea-level change (Fig. 2b, Supplementary Fig. 2), which resulted in contrasting sea-level rising trends in the different portions of the Mediterranean basin analyzed in this study. Between −8000 and −5000 CE, faster sea-level rise rates were observed in the mid-western portion of the basin (<~10° E) with rates of 8.0 ± 0.4 mm a^−1^ (Fig. 3). During the same period, sea-level rose much slower in the eastern portion of the basin (>~10°E) with rates that did not exceed 5.0 ± 0.5 mm a^−1^ (Fig. 3). We then observed an inversion in this rising pattern after sea-level stabilized around −5000 CE. Since that period, rates of sea-level rise were always slower in the western portion of the basin (<~5°E) compared to the mid-eastern part (>~5°E, Fig. 3). These differences are particularly significant between −5000 and −2000 CE when low average rise rates (0.5 ± 0.2 mm a^−1^) are recorded in the Balearic Sea while high average rates (2.0 ± 0.3 mm a^−1^) characterize the Ionian Sea. In the last 4000 years, we observed a progressive decrease in the spatial variability of the rising with maximum average rates (0.8 ± 0.2 mm a^−1^) still recorded in the Ionian Sea, while minimal average rates (0.2 ± 0.2 mm a^−1^) are recorded both in the Balearic Sea and in the Gulf of Gabes (Fig. 3). We remark that the Gulf of Gabes, in Tunisia, represents a unique setting within the Mediterranean, as it is the only Mediterranean region where a purely isostatic mid-Holocene highstand is recorded, mediated by continental levering effects^27^.

Sediment compaction and tectonics may have a role in controlling the observed spatial variability of sea-level change rates among regions. However, these components were minimized in our SLIPs database by prioritizing samples that are virtually incompressible or less prone to compaction, and by excluding data from regions that are significantly affected by co-seismic or volcano-tectonic vertical ground motions (see “Methods”). For this reason, much of the observed spatial variability of sea-level change rates is related to glacio- and hydro-isostatic adjustment (GIA), which has been the dominant process influencing the Mediterranean RSL evolution since the global mean sea-level stabilization of the mid-Holocene^7,23^. Our data indicate a general eastward increase of the GIA component in the central and western Mediterranean basin with minimal isostatic-driven subsidence recorded along the Spanish coast and in the Balearic Islands and maximum rates recorded in the Ionian Sea. This pattern differs from the one proposed by the available GIA models^7,28^ which predict the maximal GIA-related sea-level change, with comparable magnitude, in the Ionian Sea and in the area comprising the Balearic, Sardinia and Corsica islands (Supplementary Fig. 3). The offset between the data and models is probably related to lateral variations in mantle viscosity and/or in the thickness of the lithosphere, which are currently not taken into account by Mediterranean GIA models^15,29^. Lateral heterogeneity of the Earth’s mantle may significantly affect the Earth’s response to deglaciation^30,31^. Our results can thus be employed for an improved tuning of geophysical predictions of RSL evolution in the basin, which is characterized by significant variability in lithospheric thickness and complex mantle structure^19^. Nonetheless, it should be noted that our analysis has some geographic limitations due to the absence of SLIPs along much of the African coast and near the Gibraltar Strait.

### Regional climatic influence on sea-level rise rates

Notwithstanding regional differences, our spatio-temporal analysis shows that the central and western Mediterranean regions were characterized by several sea-level oscillations in the last 6 millennia (Supplementary Fig. 2). Looking for the source of these sea-level oscillations, isostatic processes can be excluded. Isostasy is, by definition, a progressive and slow viscoelastic response of the Earth to the redistribution of ice and ocean loads^32,33^. GIA modeling is unable to resolve the scale of sea-level fluctuations observed, and the oscillatory patterns observed have a period that is too short to be influenced by glacio-isostatic processes. The fact that these fluctuations were observed across all regions would also exclude potential local tectonic influences and compaction-related subsidence. Instead, we suggest that regional climatic forcings are the most likely mechanism driving the variability in the sea-level change data.

**Fig. 4:**
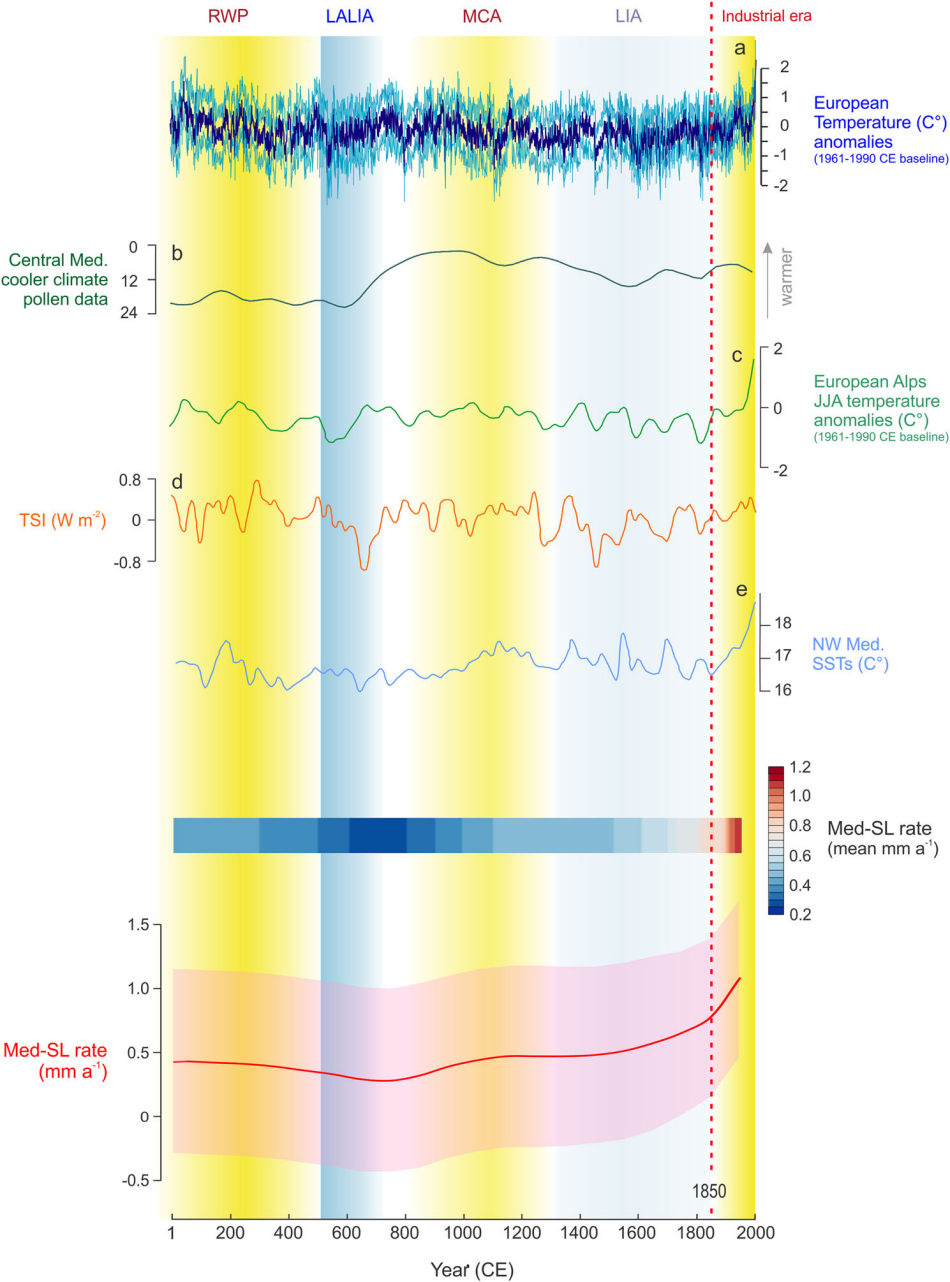
Reconstructed variability of common sea-level signal (Med-SL) for the central and western Mediterranean region in the Common Era. Solid line and shaded envelope are the model mean and 1 s uncertainty. Med-SL is compared with **a** European Temperature anomalies^37^, **b** central Mediterranean cooler climate pollen data^38^, **c** summer (JJA) temperature anomalies in the European Alps^36^, **d** total solar irradiance^41^, **e** sea-surface temperatures (SSTs) in the western Mediterranean^40^. Temporal extension of the Roman Warm Period (RWP), the Late Antique Little Ice Age (LALIA), the Medieval Climate Anomaly (MCA), the Little Ice Age (LIA), and the Industrial period are from refs. ^35,36^. The graded bar is the Med-SL model mean.

In effect, while it is known that large ice melting was minimal after −2000 CE^21,34^, much less is known about the responses to shorter-term Mediterranean climatic changes^35,36^ such as the Roman Warm Period (RWP, ~−500 CE to ~500 CE), the Late Antique Little Ice Age (LALIA, ~536 to ~660 CE), the Medieval Climate Anomaly (MCA, ~860 to ~1250 CE) and the Little Ice Age (LIA, ~1250 to ~1850 CE). In the Common Era, Med-SL rise rates varied within a range of ~0.9 mm a^−1^ (Supplementary Table 2, Fig. 4). Rise rates up to 0.5 ± 0.7 mm a^−1^ characterized the warmer episode occurring at the RWP while we observed a deceleration of the sea-level change rates (0.3 ± 0.7 mm a^−1^) during the LALIA (Fig. 4). The LALIA is recognized as one of the most important cooling episodes of the Common Era^36^. This cooling event is found in different proxies (Fig. 4) such as temperature anomalies in Europe^37^, and specifically in the central and western Mediterranean^38,39^ and the European Alps^36^. During this period, we also observe a decrease in sea-surface temperatures (SSTs) in the western Mediterranean^40^, as well as the exceptional seventh-century solar minimum^41^.

The rising trend only returned to values similar to the pre-LALIA (0.45 ± 0.7 mm a^−1^) during the MCA which was characterized by warmer climatic conditions (Fig. 4) and remained at similar values for much of the LIA (1250–1600 CE) suggesting a negligible influence of this cooling episode on central and western Mediterranean sea-level rise rates. In the remaining part of the pre-industrial period (1600 and 1800 CE) rates rose to 0.6 ± 0.6 mm a^−1^ while we observed a progressive acceleration of sea-level rise in the industrial-era, with rates up to 1.05 ± 0.6 mm a^−1^ (Fig. 4), which are significantly faster than any warm climatic episode of the Common Era.

Our spatio-temporal analysis shows a strong relationship between Mediterranean temperatures and the rate of sea-level rise confirming, at the basin scale, the results locally obtained in the northwestern Adriatic Sea^42^. These oscillations are thus controlled by the differential response of Mediterranean sea-level to cooling/warming episodes, as demonstrated by the variability of sea-level rise rates observed in the Common Era.

Therefore, our findings suggest that a deeper exploration of the regional climatic parameters, controlling the variability of rise rates, is required to produce robust predictions of Mediterranean sea-level evolution in a changing climate. Regionally, global predictions of sea-level rise may be worsened by the expected increased warming of the Mediterranean^9^. This may have major implications for the near-future resilience of both natural and human-modified Mediterranean coasts, characterized by a narrow littoral area with high concentrations of people and assets and by rapid demographic, social, economic, and environmental change^43,44^.

## Methods

### SLIPs database

We collated a database of SLIPs following the most recent standards in sea-level studies^18,45^. The analysis of a wide range of geological and archeological proxies^46–133^ resulted in a database of 401 SLIPs (Supplementary Table 1) that identify the position of former RSL in the central and western Mediterranean coasts from 12 ka to present (Supplementary Fig. 1b). Mediterranean SLIPs are commonly derived from cores on coastal and alluvial plains, coastal marshes, and lagoons. For these samples, the definition of the indicative meaning is based on paleoecology and, in particular, on the macro-and micro-faunal assemblages (i.e., malacofauna, foraminifera, and ostracod assemblages). Furthermore, fossil intertidal bioconstructions (e.g., vermetids and *Lithophyllum byssoides* rims) and beachrocks have also yielded SLIPs for the Mediterranean region^15,16^. Finally, we produced SLIPs using maritime archeological structures whose functioning height is related to the former mean sea level such as fishtanks^20^ or when found covered by fossil biological encrustations which clearly define the relationship with the former tidal frame^134^. We did not include samples from sectors manifesting major evidence for co-seismic land-level changes and/or crustal movements controlled by volcanic activity^135,136^, which can generate significant departures from climatic-driven sea-level changes. Sediment compaction can be an important issue because it can lower the SLIP relative to the initial depositional elevation, resulting in an overestimation of the sea-level rise^18,137^. In the database compilation, we prioritized “base of basal” samples, e.g., those recovered from sedimentary units overlying incompressible substrates such as Pleistocene sands/gravels or rocky basements. These samples are, therefore, less prone to compaction^6^. Where basal samples were not available, we used intercalated samples which are derived from facies of low-density, organic-rich sediment within a sequence of higher density, clastic units^18,137^, and are, therefore, susceptible to compaction. However, in the presence of two or more coeval intercalated SLIPs in the same region, we excluded those found at lower elevation as being affected by post-depositional compaction^137^. This procedure was of particular importance for the Rhone, Ebro, and Tiber deltas, for Venice lagoon, and for the Romagna and Versilia coastal plains. This practice allowed us to significantly reduce the effects of compaction on the SLIPs dataset. The fossil intertidal bioconstructions, beachrocks, and archeological structures are not subject to compaction. To perform a high-resolution assessment of variability in sea-level rise rates we clustered the data into 48 sub-regions, based on SLIPs collected no more than ~50 km apart (Supplementary Fig. 1a).

### Spatio-temporal statistical model

We employed an empirical spatio-temporal hierarchical model^16^ to reconstruct the common sea-level change across the central and western Mediterranean Basin, sub-regional variability in RSL changes, and spatial-temporal variability patterns in rates of RSL change across the basin over the last 10 ka. The height and timing (with vertical and temporal errors) of paleo-RSL from the 401 SLIPs from 48 sub-regions in the central and western Mediterranean database were fed into the model. Additional model inputs came from tide-gauge records taken from the Permanent Service for Mean Sea Level (PSMSL) (see Kopp et al.^3^ for more details). Spatio-temporal variabilities of RSL change and their uncertainties (Fig. 3) are calculated through a linear transformation of the RSL predictions.

The hierarchical model has three levels: (i) a process level, which models RSL through space and time; (ii) a data level, which models how RSL from the process model is recorded by geological proxies; and (iii) a hyperparameter level, which describes the prior expectations for spatial and temporal RSL variability.

The process model represents the RSL field as the sum of three components, each with a Gaussian Process (GP) prior:

1 $$f\left(x,t\right)={g}_{b}(t)+{r}_{s}\left(x,t\right)+{r}_{f}(x,t)$$

where ***x*** represents a spatial location and ***t*** represents time. The three components that comprise the RSL field are *g*_*b*_, which is the common Med-SL signal; *r*_*s*_(*x, t*), which represents the sub-regionally varying, slow, and temporally non-linear SL field; and *r*_*f*_(*x, t*), which represents the sub-regionally varying, fast and temporally non-linear SL field. As in Kopp et al.^3^, the data model represents observations (***y***_***i***_) as:

2 $${y}_{i}=f\left({x}_{i},{t}_{i}\right)+w\left({x}_{i},{t}_{i}\right)+{y}_{0}\left({x}_{i}\right)+{\varepsilon }_{i}^{y}\,$$

where *x*_*i*_ and *t*_*i*_ are the location and time, respectively, of observation *i*, *w*(*x, t*) is a white noise term that captures sub-decadal changes in RSL and vertical errors beyond those nominally represented in the database, and *y*_*o*_(*x*_*i*_) is a site-specific datum offset. ${\varepsilon }_{i}^{y}$ represents errors in sea-level observations, and the term for time ${t}_{i}$ is the sum of the mean observed age and an error term for time. As in Kopp et al.^3^, geochronological uncertainties are incorporated using the noisy-input GP method^138^, which translates errors in the independent variable into comparable errors in the dependent variable.

Hyperparameters that define prior knowledge of the amplitude and spatio-temporal scales for sea-level in each term of the process model were optimized via maximum likelihood. Optimized prior standard deviations for the common, slow, fast, and white noise terms were 10.4 m, 1.72 m, 2 cm, and 7 mm, respectively. The optimized temporal scales for the common, slow, and fast components were 12200, 3590, and 14.1 years, respectively, while the optimized spatial scales for the slow and fast components were 14° and 2° angular distances, respectively.

## Supplementary information

Supplementary Information

## Data Availability

Data related to this article can be found in Supplementary Table 1 (SLIP database) and Supplementary Table 2 (Med-SL rates). The references of the original papers used to produce the SLIP database are provided at the end of Supplementary Table 1. These data are available under a CC-BY 4.0 license at the following 10.5281/zenodo.4737120.

## Code availability

The code used for analysis in this article is archived on GitHub (https://github.com/bobkopp/CESL-STEHM-GP).
