# Supplementary material for: Climate pacing of millennial sea-level change variability in the central and western Mediterranean
Source: Nat Commun. 2021 Jun 29;12:4013. doi: 10.1038/s41467-021-24250-1 (PMC8242029; doi:10.1038/s41467-021-24250-1)
Supplement: Supplementary file 1 — Supplementary Information [file 41467_2021_24250_MOESM1_ESM.pdf]

Climate pacing of millennial sea-level change variability in the central and western Mediterranean

Supplementary figures

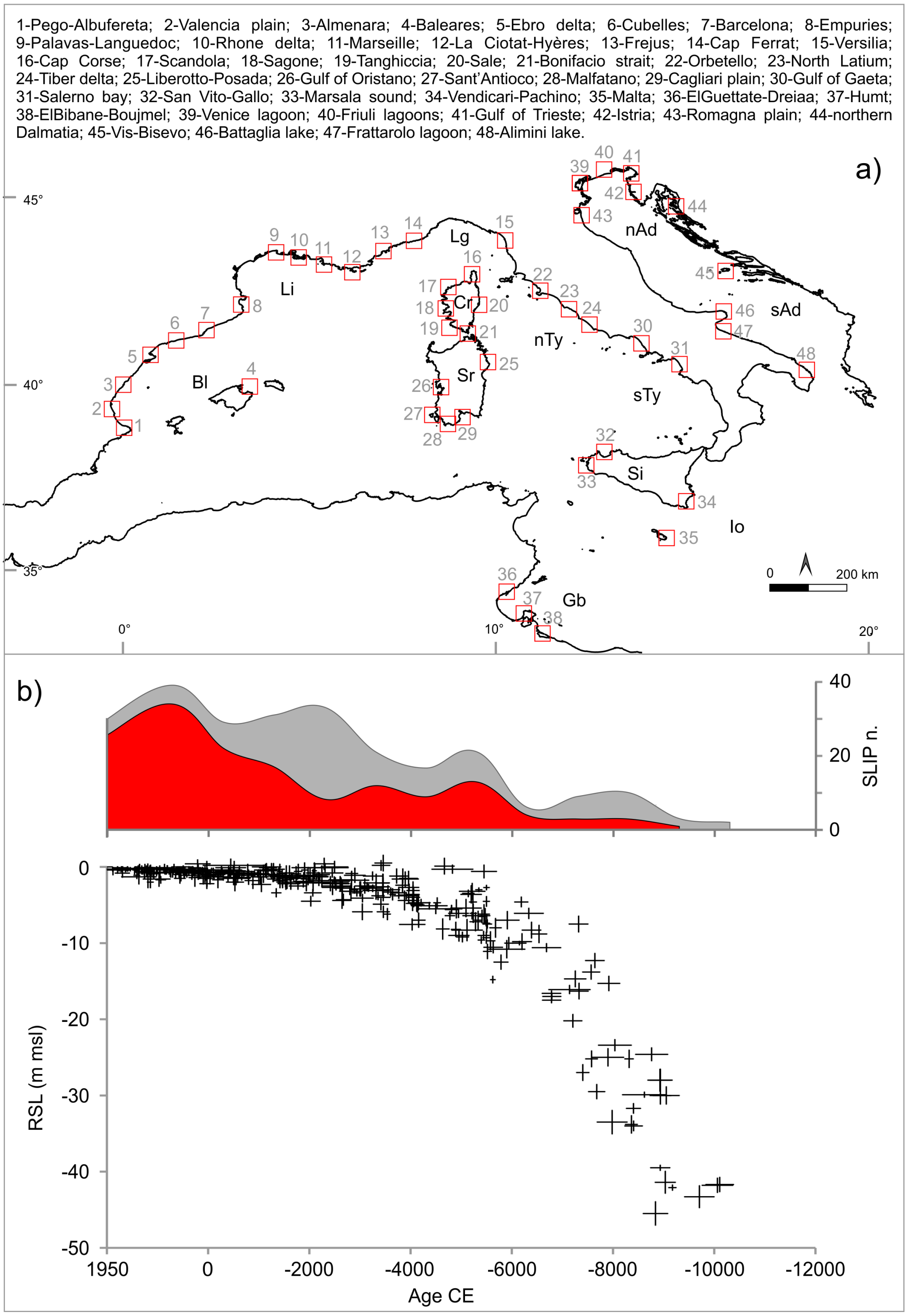

Supplementary Fig. 1. a) Geographical distribution of the 48 regions included in the study. b) Total plot and temporal distribution of the 401 Sea Level Index Points (SLIPs) used for the Relative Sea Level (RSL) reconstructions in the 48 regions. Grey area indicates the cumulative curve of the intercalated SLIPs while red area indicates the cumulative curve of those SLIPs less prone to compactions (base of basal samples) or virtually incompressible (fixed biological, beachrocks, and archaeological indicators).

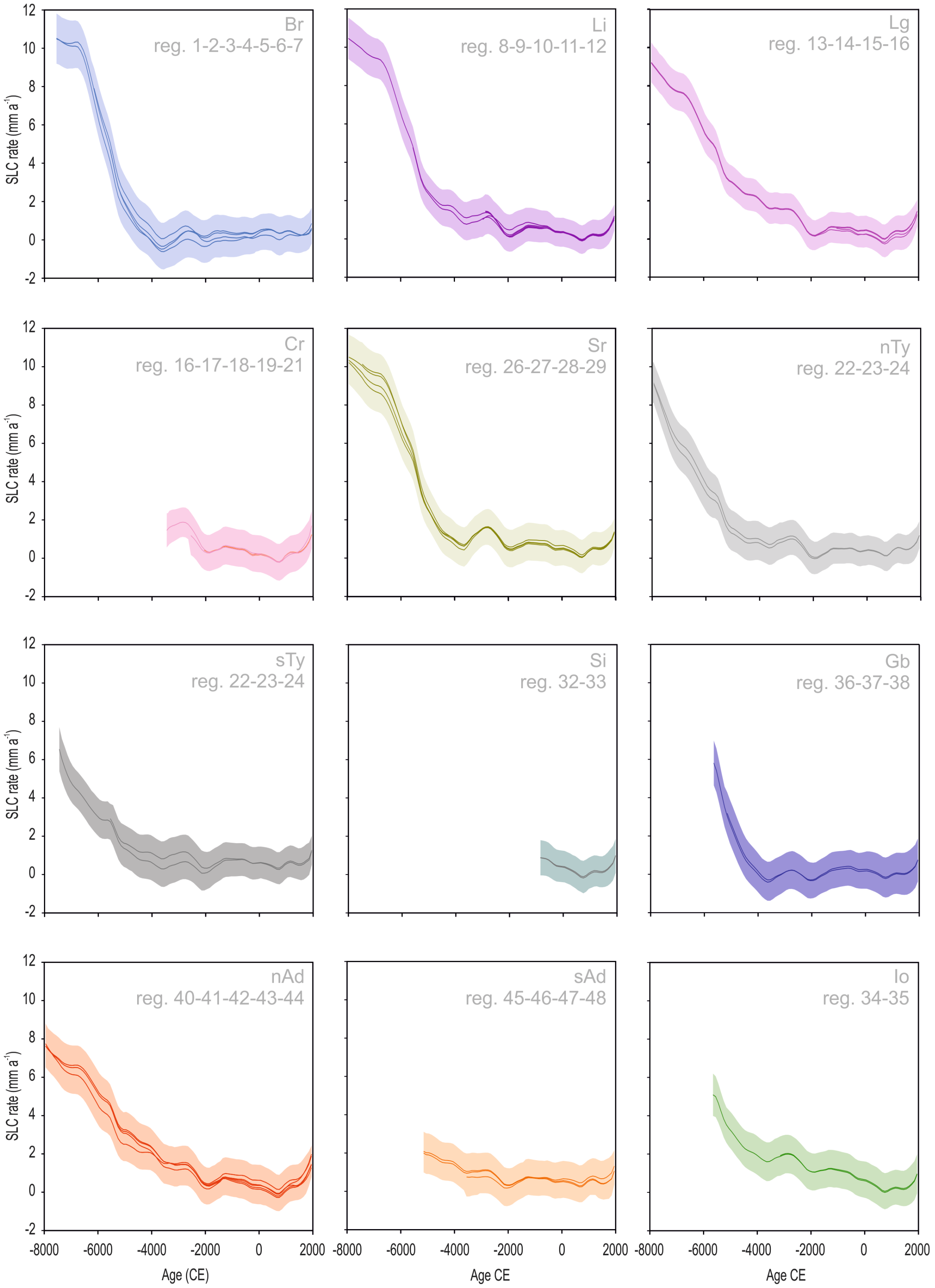

Supplementary Fig. 2. Variability of Relative Sea Level (RSL) change rates in the 48 central and western Mediterranean regions since -8000 CE. The solid line and shaded envelope are the model mean and 1s uncertainty. The regions are grouped according to geographical position. Br is the Balearic Sea. Li is the Gulf of Lion. Lg is the Ligurian Sea. Cr is Corsica. Sr is Sardinia. Ty is the northern Tyrrhenian Sea. sTy is the southern Tyrrhenian Sea. Si is Sicily. Gb is the Gulf of Gabes. nAd is the northern Adriatic. sAd is the southern Adriatic. Io is the Ionian Sea. For the region numbers please refer to Supplementary Fig. 1

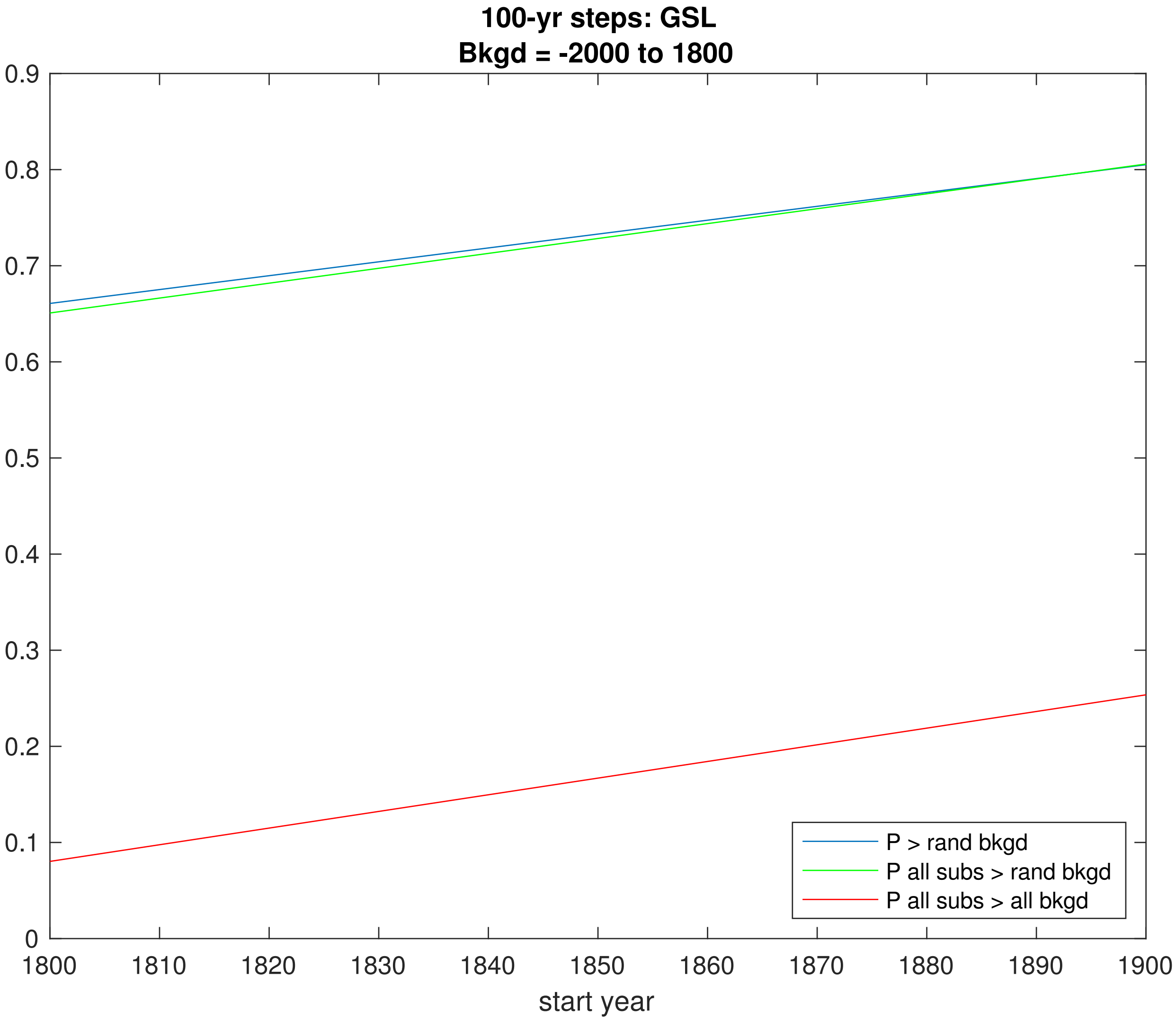

Supplementary Fig. 3. Probabilities that minimum rates within subsequent 100-year time periods (1800 to 1900 CE and 1900 to 2000 CE) will exceed rates from the background period (-2000 to 1800 CE). Blue curve shows probabilities that minimum rates from subsequent periods will exceed random background rates. Green curve shows probabilities that all minimum rates from subsequent periods will exceed random background rates. Red curve shows probabilities that all minimum rates from subsequent periods will exceed all background rates.

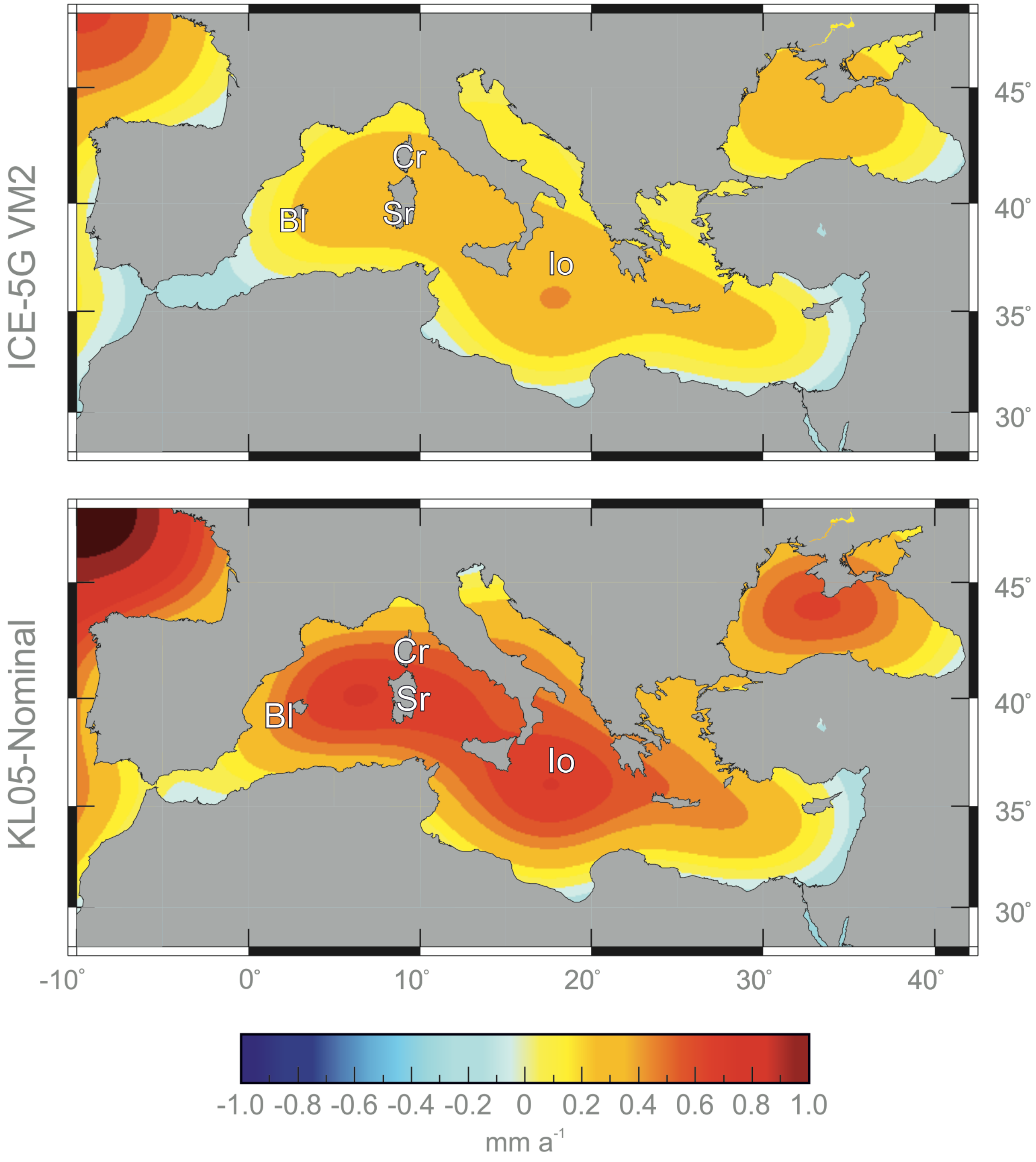

Supplementary Fig. 4. Modelled Glacio and hydro-isostatic adjustment (GIA) sea-level fingerprints in mm a-1for 2040-2050 relative to 1990-2000 in the Mediterranean Sea [1; 2]. Predictions were obtained using models ICE-5G (VM2) [3] and KL05 [4]. Bl is Balearic islands. Sr is Sardinia Island. Cr is Corsica Island. Io is Ionian Sea.

Supplementary Tables

Supplementary table 1. Mediterranean Sea Level Index Points (SLIPs) used for the spatio-temporal analysis and the original data source. Latitudes and longitudes are expressed to two decimal degrees of precision. Age are expressed in calibrated years before of after Common Era. Upper error bars (+) are forced to present msl with the sole exception of the Gulf of Gabes which is the single location characterized by a non-tectonic highstand above the present msl in the central and western Mediterranean [5]. SLIPs in bold are less prone to compaction or virtually incompressible (e.g.. base of basal. fixed biological indicators. beachrocks and archaeological markers). SLIPs extracted from the [5] database are marked with *. SLIPs extracted from the [1] database are marked with **. Full references are provided at the end of the table.

Site	*Reg.*	*Lat.*	*Long.*	*Lab code*	*RSL (m)*	*Error m (+)*	*Error m (-)*	*Age CE*	*Error*	*Material*	*Reference*	
Pego-Albufereta	1	38.36	-0.44	Beta-213544	-1.4	1.0	1.0	-850	53	Peat	6	*
Pego-Albufereta	1	38.36	-0.44	Beta-213540	-0.3	0.3	0.6	757	103	Peat	6	*
**Pego-Albufereta**	**1**	**38.87**	**0.08**	**Beta-46429**	**-3.0**	**1.1**	**1.1**	**-5401**	**71**	**Organics**	**7**	
Pego-Albufereta	1	38.86	0.05	Poz-93699	-10.0	1.1	1.1	-6202	141	Organics	7	
Pego-Albufereta	1	38.82	0.03	Poz-93698	-16.6	1.1	1.1	-6836	192	Organics	7	
Pego-Albufereta	1	38.82	0.03	Poz-93965	-17.0	1.1	1.1	-6842	187	Organics	7	
Pego-Albufereta	1	38.82	0.03	Poz-93966	-17.5	1.1	1.1	-6838	190	Organics	7	
Valencia Plain	2	38.87	-0.06	UBAR-44	-10.6	0.6	0.6	-6737	291	Peat	8	*
Valencia Plain	2	38.87	-0.06	UBAR-78	-16.1	0.6	0.6	-7192	417	Peat	8	*
Valencia Plain	2	39.15	-0.25	Beta-147301	-1.8	1.1	1.1	-2885	351	Shell	9	*
Valencia Plain	2	39.30	-0.32	Beta-215488	-0.9	0.6	0.6	-1513	95	Pollen	10	*
Valencia Plain	2	39.30	-0.32	Beta-208373	-3.5	0.5	0.5	-5192	122	Pollen	10	*
Valencia Plain	2	39.30	-0.32	Beta204241	-4.5	0.7	0.7	-5550	69	Pollen	10	*
Valencia Plain	2	39.34	-0.36	na	-1.3	1.0	1.0	1318	349	Shell	11	*
**Valencia Plain**	**2**	**39.45**	**-0.33**	**R-2176**	**-6.1**	**0.7**	**0.7**	**-6388**	**295**	**Peat**	**12**	*
Valencia Plain	2	39.45	-0.33	R-2168	-2.1	0.7	0.7	-2564	266	Peat	9	*
Valencia Plain	2	39.75	-0.18	Beta-386336	-1.1	0.2	0.2	-83	86	Organics	13	**
Valencia Plain	2	39.39	-0.37	Beta-346465	-0.5	0.5	0.5	1132	90	Plants	14	**
Valencia Plain	2	39.39	-0.37	Beta-377886	-1.0	0.5	0.5	592	53	Seeds	14	**
Valencia Plain	2	39.39	-0.37	Beta-346466	-1.5	0.5	0.5	-444	218	Shell	14	**
Valencia Plain	2	39.39	-0.37	Beta-346467	-2.5	0.5	0.5	-1826	180	Shell	14	**
Valencia Plain	2	39.39	-0.37	Beta-362537	-2.7	1.0	1.0	-2509	214	Shell	14	**
Valencia Plain	2	39.39	-0.37	Beta-346464	-2.8	1.0	1.0	-2702	174	Shell	14	**
**Valencia Plain**	**2**	**39.39**	**-0.37**	**Beta-346468**	**-4.6**	**0.7**	**0.7**	**-6239**	**140**	**Organics**	**14**	**
Almenara	3	39.75	-0.18	Beta-288410	-0.9	0.3	0.3	-2889	127	Organics	14	**

Site	*Reg.*	*Lat.*	*Long.*	*Lab code*	*RSL (m)*	*Error m (+)*	*Error m (-)*	*Age CE*	*Error*	*Material*	*Reference*	
Almenara	3	39.75	-0.18	Beta-288412	-2.7	0.3	0.3	-5552	65	Organics	15	**
Baleares	4	39.83	3.11	Poz-72248	-0.2	0.2	1.0	969	162	Shell	16	**
Baleares	4	39.83	3.11	Poz-69098	-0.2	0.2	0.6	624	145	Shell	16	**
Baleares	4	39.83	3.11	Poz-69459	-0.8	0.6	0.6	369	174	Shell	16	**
Baleares	4	39.83	3.11	Poz-72246b	-0.4	0.4	1.2	-26	166	Shell	16	**
Baleares	4	39.83	3.11	Beta-69334	-2.0	1.2	1.2	-582	178	Organics	16	**
Baleares	4	39.83	3.11	Poz-69099	-0.5	0.5	1.0	-1205	188	Shell	16	**
Baleares	4	39.83	3.11	Poz-69465	-0.5	0.5	1.0	-1302	179	Shell	16	**
Baleares	4	39.83	3.11	Poz-69460	-0.6	0.6	1.0	-1468	52	Plant remains	16	**
Baleares	4	39.83	3.11	Poz-69461	-0.8	0.8	1.0	-1597	138	Plant remains	16	**
Baleares	4	39.83	3.11	Poz-69454	-0.9	0.9	1.0	-1790	98	Plant remains	16	**
Baleares	4	39.83	3.11	Poz-69453	-0.6	0.6	1.0	-1832	84	Plant remains	16	**
Baleares	4	39.83	3.11	Poz-69541	-0.4	0.4	1.0	-1958	70	Plant remains	16	**
Baleares	4	39.83	3.11	Poz-69455	-1.1	1.0	1.0	-2066	121	Plant remains	16	**
Baleares	4	39.83	3.11	Poz-69470	-0.8	0.6	0.6	-2504	220	Shell	16	**
Baleares	4	39.83	3.11	Poz-69466	-1.1	1.0	1.0	-2950	213	Shell	16	**
Baleares	4	39.83	3.11	Poz-69472	-1.2	0.6	0.6	-3301	192	Charcoal	16	**
**Baleares**	**4**	**39.83**	**3.11**	**Poz-72246**	**-1.2**	**1.0**	**1.0**	**-3776**	**146**	**Shell**	**16**	**
Baleares	4	39.83	3.11	Poz-69473	-1.2	1.0	1.0	-3899	121	Charcoal	16	**
**Baleares**	**4**	**39.83**	**3.11**	**Poz-69467b**	**-1.6**	**1.0**	**1.0**	**-3947**	**267**	**Shell**	**16**	**
**Baleares**	**4**	**39.83**	**3.11**	**Poz-69467**	**-1.5**	**1.0**	**1.0**	**-4012**	**184**	**Shell**	**16**	**
Baleares	4	39.93	3.95	Ua-2729	-5.4	1.2	1.2	-5146	310	Shell	17	**
Baleares	4	39.83	3.11	ETH-11904	-3.6	1.2	1.2	-5248	213	Organics	18	**
Baleares	4	39.83	3.11	Ua-2732	-7.0	1.2	1.2	-5962	242	Organics	18	**
Ebro delta	5	40.7	0.60	Beta-380019	-0.1	0.1	0.6	469	69	Shell	19	**
Ebro delta	5	40.7	0.60	Beta-380020	-0.4	0.4	0.6	441	95	Shell	19	**
Ebro delta	5	40.7	0.60	Beta-348514	-0.5	0.5	0.6	330	77	Shell	19	**
Ebro delta	5	40.7	0.60	Beta-348515	-0.6	0.6	0.6	242	103	Shell	19	**
Ebro delta	5	40.7	0.60	Beta-380021	-0.8	0.6	0.6	-672	126	Shell	19	**
Ebro delta	5	40.7	0.60	Beta-348516	-0.9	0.6	0.6	-645	136	Shell	19	**
Cubelles	6	41.20	1.66	GD-5918	-0.5	0.5	0.7	1101	104	Organics	20	**
Cubelles	6	41.20	1.66	GD-6597	-0.1	0.1	1.2	-498	305	Organics	20	**
Cubelles	6	41.20	1.66	GD-7041	-1.2	1.2	1.2	-2025	244	Organics	20	**
Cubelles	6	41.20	1.66	GD-5919	-3.4	0.8	0.8	-3818	151	Organics	20	**
Barcelona	7	41.33	2.05	Poz-19098	-3.0	0.5	0.5	-2943	68	Organics	21	**

Site	*Reg.*	*Lat.*	*Long.*	*Lab code*	*RSL (m)*	*Error m (+)*	*Error m (-)*	*Age CE*	*Error*	*Material*	*Reference*	
Barcelona	7	41.33	2.05	Poz-19180	-2.7	0.5	0.5	-4022	199	Organics	21	**
Barcelona	7	41.33	2.05	Poz-19247	-3.4	0.5	0.5	-2991	98	Organics	21	**
Empuries	8	42.28	3.10	Beta-272094	-0.6	0.5	0.5	1212	108	Shell	22	**
Empuries	8	42.28	3.10	Beta-272095	-0.8	0.8	1.0	905	122	Shell	22	**
Empuries	8	42.28	3.10	Beta-286856	-1.3	1.0	1.0	45	144	Shell	22	**
Empuries	8	42.28	3.10	Beta-272096	-1.4	1.0	1.0	-603	161	Shell	22	**
Empuries	8	42.28	3.10	Beta-286857	-2.6	0.5	0.5	-2687	184	Plant remains	22	**
Empuries	8	42.28	3.10	Beta-272097	-3.2	0.5	0.5	-2852	156	Plant remains	22	**
**Empuries**	**8**	**42.04**	**3.22**	**LGQ-775**	**-0.4**	**0.3**	**0.3**	**-327**	**161**	***L. byssoides***	**23**	*
Palavas-Languedoc	9	43.50	3.86	na	0.0	0.0	0.6	1489	130	Shell	24	*
Palavas-Languedoc	9	43.50	3.86	na	-0.3	0.3	0.6	1308	105	Shell	24	*
Palavas-Languedoc	9	43.52	3.88	na	-0.4	0.4	1.0	652	125	Shell	24	*
Palavas-Languedoc	9	43.52	3.88	na	0.0	0.0	1.0	409	151	Shell	24	*
Palavas-Languedoc	9	43.52	3.88	na	-0.6	0.6	1.0	-835	130	Shell	24	*
Palavas-Languedoc	9	43.52	3.88	na	-1.3	1.0	1.0	-1083	168	Shell	24	*
Palavas-Languedoc	9	43.52	3.88	na	-1.8	1.0	1.0	-1471	151	Shell	24	*
**Palavas-Languedoc**	**9**	**43.52**	**3.88**	**na**	**-2.4**	**1.0**	**1.0**	**-3892**	**141**	**Shell**	**24**	*
**Palavas-Languedoc**	**9**	**43.50**	**3.86**	**na**	**-6.4**	**1.0**	**1.0**	**-5526**	**102**	**Shell**	**24**	*
RhoneDelta	10	43.45	4.89	LY-8447	-0.1	0.1	0.5	810	140	Peat	25	*
**RhoneDelta**	**10**	**43.45**	**4.89**	**LY-8731**	**-2.8**	**0.5**	**0.5**	**-3304**	**201**	**Peat**	**25**	*
**RhoneDelta**	**10**	**43.45**	**4.89**	**LY-8621**	**-3.1**	**0.5**	**0.5**	**-3508**	**132**	**Peat**	**25**	*
**RhoneDelta**	**10**	**43.45**	**4.89**	**LY-7081**	**-3.8**	**0.5**	**0.5**	**-3967**	**255**	**Peat**	**25**	*
**RhoneDelta**	**10**	**43.45**	**4.89**	**LY-8446**	**-4.7**	**0.5**	**0.5**	**-4259**	**189**	**Peat**	**25**	*
**RhoneDelta**	**10**	**43.45**	**4.89**	**LY-364**	**-5.1**	**0.5**	**0.5**	**-4421**	**362**	**Peat**	**25**	*
**RhoneDelta**	**10**	**43.45**	**4.89**	**LY-8218**	**-5.5**	**0.5**	**0.5**	**-4863**	**135**	**Peat**	**25**	*
**RhoneDelta**	**10**	**43.45**	**4.89**	**LY-8671**	**-6.4**	**0.5**	**0.5**	**-5266**	**191**	**Peat**	**25**	*
**RhoneDelta**	**10**	**43.49**	**4.52**	**LY-8681**	**-7.4**	**0.4**	**0.4**	**-5546**	**70**	**Peat**	**25**	*
RhoneDelta	10	43.45	4.38	na	-33.8	1.2	1.2	-8415	127	Organics	26	*
RhoneDelta	10	43.52	4.33	na	-31.7	0.7	0.7	-8455	147	Organics	26	*

Site	***Reg.***	***Lat.***	***Long.***	***Lab code***	***RSL (m)***	***Error m (+)***	***Error m (-)***	***Age CE***	***Error***	***Material***	***Reference***	
RhoneDelta	10	43.49	4.52	MC-2017	-29.9	0.4	0.4	-8671	439	Peat	28	*
RhoneDelta	10	43.49	4.52	POZ-3937	-39.5	0.4	0.4	-8984	200	Peat	28	*
RhoneDelta	10	43.45	4.38	na	-41.4	1.5	1.5	-9084	207	Organics	26	*
RhoneDelta	10	43.49	4.52	POZ-3938	-42.1	0.4	0.4	-9226	81	Peat	28	*
**RhoneDelta**	**10**	**43.45**	**4.38**	**na**	**-43.3**	**1.5**	**1.5**	**-9757**	**301**	**wood**	**26**	*
Marseille	11	43.29	5.37	LGQ 906	-0.2	0.2	0.3	1639	195	Shell	27	*
Marseille	11	43.29	5.37	MC-697 A	-0.4	0.3	0.3	87	302	Shell	27	*
Marseille	11	43.29	5.37	MC-697 B	-0.4	0.3	0.3	-36	299	Shell	27	*
**Marseille**	**11**	**43.29**	**5.37**	**na**	**-0.4**	**0.3**	**0.3**	**500**	**50**	**Shell on Roman quay**	**27**	*
**Marseille**	**11**	**43.29**	**5.37**	**na**	**-0.7**	**0.3**	**0.3**	**-475**	**25**	**Shell on Archaic quay**	**27**	*
**Marseille**	**11**	**43.29**	**5.37**	**na**	**-0.8**	**0.3**	**0.3**	**-475**	**25**	**Shell on wooden post**	**27**	*
**Marseille**	**11**	**43.29**	**5.37**	**LY-9008**	**-0.8**	**0.3**	**0.3**	**-831**	**265**	**Shell**	**27**	*
**Marseille**	**11**	**43.29**	**5.37**	**LY-8374**	**-1.5**	**0.3**	**0.3**	**-1742**	**258**	**Mesophyllum**	**27**	*
**Marseille**	**11**	**43.29**	**5.37**	**LY-8423**	**-1.7**	**0.3**	**0.3**	**-2676**	**247**	**Shell**	**27**	*
**Marseille**	**11**	**43.29**	**5.37**	**-**	**-0.2**	**0.3**	**0.3**	**1660**	**50**	**Shell on ditch**	**27**	*
**La Ciotat-Hyères**	**12**	**43.16**	**5.58**	**LGQ-802**	**-0.2**	**0.2**	**0.4**	**1332**	**78**	***L. byssoides***	**23**	*
**La Ciotat-Hyères**	**12**	**43.16**	**5.58**	**LGQ-801**	**-0.3**	**0.3**	**0.4**	**1098**	**114**	***L. byssoides***	**23**	*
**La Ciotat-Hyères**	**12**	**43.16**	**5.58**	**LGQ-799**	**-0.3**	**0.3**	**0.4**	**1391**	**94**	***L. byssoides***	**23**	*
**La Ciotat-Hyères**	**12**	**43.16**	**5.58**	**LGQ-800**	**-0.4**	**0.4**	**0.4**	**669**	**105**	***L. byssoides***	**23**	*
**La Ciotat-Hyères**	**12**	**43.16**	**5.58**	**LGQ-797**	**-0.4**	**0.4**	**0.4**	**1372**	**75**	***L. byssoides***	**23**	*
**La Ciotat-Hyères**	**12**	**43.16**	**5.58**	**LGQ-798**	**-0.4**	**0.4**	**0.4**	**826**	**143**	***L. byssoides***	**23**	*
**La Ciotat-Hyères**	**12**	**43.16**	**5.58**	**LGQ-760**	**-1.1**	**0.4**	**0.4**	**-1695**	**197**	***L. byssoides***	**23**	*
**La Ciotat-Hyères**	**12**	**43.16**	**5.58**	**LGQ-763**	**-1.3**	**0.4**	**0.4**	**-1713**	**179**	***L. byssoides***	**23**	*
**La Ciotat-Hyères**	**12**	**43.16**	**5.58**	**LGQ-761**	**-1.3**	**0.4**	**0.4**	**-1463**	**162**	***L. byssoides***	**23**	*
**La Ciotat-Hyères**	**12**	**43.16**	**5.58**	**LGQ-773**	**-0.2**	**0.2**	**0.4**	**1167**	**122**	***L. byssoides***	**23**	*

Site	*Reg.*	*Lat.*	*Long.*	*Lab code*	*RSL (m)*	*Error m (+)*	*Error m (-)*	*Age CE*	*Error*	*Material*	*Reference*	
**La Ciotat-Hyères**	**12**	**43.16**	**5.58**	**LGQ-769**	**-0.5**	**0.4**	**0.4**	**439**	**170**	***L. byssoides***	**23**	*
**La Ciotat-Hyères**	**12**	**43.16**	**5.58**	**LGQ-768**	**-0.9**	**0.4**	**0.4**	**-618**	**188**	***L. byssoides***	**23**	*
**La Ciotat-Hyères**	**12**	**43.16**	**5.58**	**LGQ-764**	**-1.0**	**0.4**	**0.4**	**-1028**	**182**	***L. byssoides***	**23**	*
**La Ciotat-Hyères**	**12**	**43.16**	**5.58**	**LGQ-770**	**-1.0**	**0.4**	**0.4**	**-1088**	**177**	***L. byssoides***	**23**	*
**La Ciotat-Hyères**	**12**	**43.16**	**5.58**	**LGQ-765**	**-1.3**	**0.4**	**0.4**	**-2194**	**236**	***L. byssoides***	**23**	*
**La Ciotat-Hyères**	**12**	**43.16**	**5.58**	**LGQ-766**	**-1.3**	**0.4**	**0.4**	**-1435**	**172**	***L. byssoides***	**23**	*
**La Ciotat-Hyères**	**12**	**43.16**	**5.58**	**LGQ-767**	**-1.4**	**0.4**	**0.4**	**-1689**	**187**	***L. byssoides***	**23**	*
**La Ciotat-Hyères**	**12**	**43.03**	**6.10**	**LGQ-682**	**-0.5**	**0.4**	**0.4**	**268**	**137**	***L. byssoides***	**23**	*
**La Ciotat-Hyères**	**12**	**43.03**	**6.10**	**LGQ-683**	**-0.7**	**0.4**	**0.4**	**-244**	**151**	***L. byssoides***	**23**	*
**La Ciotat-Hyères**	**12**	**43.03**	**6.10**	**LGQ-684**	**-0.9**	**0.4**	**0.4**	**-477**	**272**	***L. byssoides***	**23**	*
**La Ciotat-Hyères**	**12**	**43.01**	**6.38**	**LGQ-829**	**-0.9**	**0.4**	**0.4**	**-907**	**112**	***L. byssoides***	**23**	*
**La Ciotat-Hyères**	**12**	**43.01**	**6.38**	**LGQ-826**	**-1.1**	**0.4**	**0.4**	**-720**	**180**	***L. byssoides***	**23**	*
Frejus	13	43.43	6.70	Esc-1.19	-0.5	0.6	0.6	-670	126	Grains	29	*
**Frejus**	**13**	**43.43**	**6.73**	**POZ-14371**	**-0.4**	**0.3**	**0.3**	**-119**	**146**	**Shell**	**30**	*
**Frejus**	**13**	**43.43**	**6.73**	**POZ-14372**	**-0.4**	**0.3**	**0.3**	**-155**	**151**	**Shell**	**30**	*
**Frejus**	**13**	**43.43**	**6.73**	**POZ-24339**	**-0.4**	**0.3**	**0.3**	**507**	**111**	**Shell**	**31**	*
**Frejus**	**13**	**43.43**	**6.73**	**LY-9154**	**-0.3**	**0.3**	**0.3**	**564**	**106**	**Shell**	**31**	*
**Frejus**	**13**	**43.43**	**6.73**	**-**	**-0.5**	**0.2**	**0.2**	**35**	**35**	**shells on fishtank**	**31**	*
**Frejus-Dramont**	**13**	**43.41**	**6.85**	**LGQ- 703**	**-0.5**	**0.4**	**0.4**	**-473**	**272**	***L. byssoides***	**23**	*
**Frejus-Dramont**	**13**	**43.41**	**6.85**	**LGQ- 697**	**-0.9**	**0.4**	**0.4**	**-967**	**153**	***L. byssoides***	**23**	*
**Cap-Ferrat**	**14**	**43.67**	**7.32**	**LGQ- 859**	**-0.2**	**0.4**	**0.4**	**755**	**122**	***L. byssoides***	**23**	*
Versilia	15	43.83	10.33	Poz-11380	-4.8	1.0	1.0	-4090	132	Charcoal	32	*
Versilia	15	43.83	10.33	Poz-11381	-4.9	1.0	1.0	-4175	131	-	32	*
Versilia	15	43.83	10.33	Poz-11382	-5.0	1.0	1.0	-4190	136	Bark	32	*
**Versilia**	**15**	**43.83**	**10.33**	**Poz-10665**	**-6.4**	**0.6**	**0.6**	**-4829**	**103**	**Charcoal**	**32**	*
Versilia	15	43.81	10.34	na	-1.0	0.6	0.6	-255	139	Organics	33	*

Site	*Reg.*	*Lat.*	*Long.*	*Lab code*	*RSL (m)*	*Error m (+)*	*Error m (-)*	*Age CE*	*Error*	*Material*	*Reference*	
Versilia	15	43.81	10.34	na	-2.1	0.6	0.6	-2300	156	Wood	33	*
Versilia	15	43.81	10.34	na	-2.2	0.6	0.6	-2662	174	Shell	33	*
Versilia	15	43.81	10.34	na	-9.0	0.6	0.6	-5493	119	Shell	33	*
Versilia	15	43.81	10.34	na	-34.0	0.7	0.7	-8458	182	Shell	33	*
Versilia	15	43.81	10.34	na	-10.0	0.8	0.8	-5991	87	Shell	33	*
Versilia	15	43.73	10.43	S1	-12.5	1.0	1.0	-5838	147	Shell	33	*
Versilia	15	43.7	10.3	na	-4.7	1.0	1.0	-4206	206	Shell	33	*
Portus Pisanus	15	43.70	10.30	na	0.0	0.0	0.5	1258	34	Seed	34	
Portus Pisanus	15	43.70	10.30	na	-3.3	0.6	0.6	-3660	172	Shell	34	
Portus Pisanus	15	43.70	10.30	na	-2.6	0.6	0.6	-3193	154	Seed	34	
**Cap Corse**	**16**	**43.02**	**9.40**	**LGQ- 835**	**-0.3**	**0.3**	**0.4**	**967**	**188**	***L. byssoides***	**23**	*
**Cap Corse**	**16**	**43.02**	**9.40**	**LGQ- 836**	**-0.8**	**0.4**	**0.4**	**-477**	**272**	***L. byssoides***	**23**	*
**Cap Corse**	**16**	**43.02**	**9.40**	**LGQ- 837**	**-0.9**	**0.4**	**0.4**	**-758**	**211**	***L. byssoides***	**23**	*
**Cap Corse**	**16**	**43.02**	**9.40**	**LGQ- 838**	**-1.1**	**0.4**	**0.4**	**-1365**	**139**	***L. byssoides***	**23**	*
**Cap Corse**	**16**	**42.96**	**9.34**	**LGQ- 839**	**-0.4**	**0.4**	**0.4**	**316**	**188**	***L. byssoides***	**23**	*
**Cap Corse**	**16**	**42.96**	**9.34**	**LGQ- 840**	**-0.6**	**0.4**	**0.4**	**1161**	**118**	***L. byssoides***	**23**	*
**Cap Corse**	**16**	**42.96**	**9.34**	**LGQ- 841**	**-1.0**	**0.4**	**0.4**	**-1194**	**197**	***L. byssoides***	**23**	*
**Cap Corse**	**16**	**42.96**	**9.34**	**LGQ- 842**	**-1.1**	**0.4**	**0.4**	**-1940**	**193**	***L. byssoides***	**23**	*
**Cap Corse**	**16**	**42.96**	**9.34**	**LGQ- 843**	**-1.3**	**0.4**	**0.4**	**-1812**	**198**	***L. byssoides***	**23**	*
**Scandola**	**17**	**42.37**	**8.54**	**LGQ- 832**	**-0.4**	**0.4**	**0.4**	**551**	**123**	***L. byssoides***	**23**	*
**Scandola**	**17**	**42.37**	**8.54**	**LGQ- 834**	**-0.6**	**0.4**	**0.4**	**-455**	**271**	***L. byssoides***	**23**	*
**Scandola**	**17**	**42.37**	**8.54**	**LGQ- 833**	**-1.1**	**0.4**	**0.4**	**-1573**	**160**	***L. byssoides***	**23**	*
**Scandola**	**17**	**42.37**	**8.54**	**LGQ- 862**	**-1.6**	**0.4**	**0.4**	**-2041**	**158**	***L. byssoides***	**23**	*
**Scandola**	**17**	**42.37**	**8.54**	**LGQ- 861**	**-0.5**	**0.4**	**0.4**	**297**	**209**	***L. byssoides***	**23**	*
**Scandola**	**17**	**42.37**	**8.54**	**LGQ- 864**	**-0.8**	**0.4**	**0.4**	**-197**	**198**	***L. byssoides***	**23**	*
Sagone	18	42.11	8.69	Poz-65992	-0.4	0.4	0.5	-317	87	Plant remains	35	**
Sagone	18	42.11	8.69	Poz-65994	-0.4	0.4	0.5	-458	76	Plant remains	35	**
Sagone	18	42.11	8.69	Poz-65995	-0.8	0.5	0.5	-298	93	Plant remains	35	**
Sagone	18	42.11	8.69	Poz-58646	-0.3	0.3	0.5	1261	37	Plant remains	35	**
Sagone	18	42.11	8.69	Poz-58648	-0.4	0.4	0.5	634	35	Plant remains	35	**
Sagone	18	42.11	8.69	Poz-58650	-0.6	0.5	0.5	47	89	Plant remains	35	**
Sagone	18	42.11	8.69	Poz-58651	-0.7	0.5	0.5	398	134	Plant remains	35	**
**Sagone**	**18**	**42.11**	**8.69**	**Poz-65998**	**-0.4**	**0.4**	**0.5**	**1737**	**213**	**Organics**	**35**	**
Sagone	18	42.11	8.69	Poz-65996	-0.5	0.5	0.5	1246	36	Plant remains	35	**
Sagone	18	42.11	8.69	Poz-65925	-1.2	0.5	0.5	-240	119	Organics	35	**

Site	*Reg.*	*Lat.*	*Long.*	*Lab code*	*RSL (m)*	*Error m (+)*	*Error m (-)*	*Age CE*	*Error*	*Material*	*Reference*	
Sagone	18	42.11	8.69	Poz-58642	-0.6	0.5	0.5	1105	77	Wood	35	**
Sagone	18	42.11	8.69	Poz-55976	-1.7	0.5	0.5	884	105	Wood	35	**
Tanghiccia	19	41.72	8.809	Beta-450507	-2.0	0.5	0.5	-1520	121	Shell	36	**
Tanghiccia	19	41.72	8.809	Poz-82975	-1.7	1.0	1.0	-1581	76	Wood	36	**
Tanghiccia	19	41.72	8.809	Poz-56332	-1.8	1.0	1.0	-1521	87	Plant remains	36	**
Tanghiccia	19	41.72	8.809	Poz-82974	-2.0	1.0	1.0	-1587	78	Peat	36	**
Tanghiccia	19	41.72	8.809	Poz-87300	-2.0	1.0	1.0	-1684	147	Shell	36	**
Sale	20	42.09	9.52	Poz-71368	-0.1	0.1	0.5	1142	108	Shell	37	**
Sale	20	42.09	9.52	Poz-65452	-0.4	0.4	0.5	-555	160	Shell	37	**
Sale	20	42.09	9.52	Poz-65838	-0.8	0.5	0.5	-2126	142	Charcoal	37	**
Sale	20	42.09	9.52	Poz-65450	-1.2	0.5	0.5	-2229	170	Shell	37	**
Sale	20	42.09	9.52	Poz-65451	-1.2	0.5	0.5	-2260	166	Shell	37	**
Sale	20	42.09	9.52	na	-1.9	0.5	0.5	-2669	183	Charcoal	37	**
Sale	20	42.09	9.52	Poz-65453	-2.7	0.5	0.5	-3221	152	Shell	37	**
**Sale**	**20**	**42.09**	**9.52**	**Poz-65449**	**-3.8**	**0.6**	**0.6**	**-3409**	**131**	**Shell**	**37**	**
Bonifacio Strait	21	41.37	9.26	Poz-66301	-0.8	0.8	1.0	-1659	99	Organics	38	**
Bonifacio Strait	21	41.37	9.26	Poz-66301	-1.8	0.5	0.5	-2545	73	Organics	38	**
Bonifacio Strait	21	41.28	9.35	Poz-77841	-0.7	0.6	0.6	-455	77	Organics	38	**
Bonifacio Strait	21	41.28	9.35	Poz-77844	-1.3	0.6	0.6	-1414	87	Organics	38	**
Bonifacio Strait	21	41.28	9.35	Poz-77842	-0.8	0.6	0.6	1642	149	Organics	38	**
**Orbetello**	**22**	**42.43**	**11.17**	**na**	**-0.4**	**0.4**	**1.0**	**523**	**124**	**Shell**	**39**	
**Orbetello**	**22**	**42.43**	**11.15**	**-**	**-0.9**	**0.4**	**0.4**	**0**	**50**	**Fishtank**	**40; 41**	*
**S. Marinella Gr.**	**23**	**42.03**	**11.86**	**-**	**-1.0**	**0.6**	**0.6**	**0**	**50**	**Fishtank**	**40; 41**	*
**Marinella Od.**	**23**	**42.03**	**11.90**	**-**	**-0.9**	**0.5**	**0.5**	**0**	**50**	**Fishtank**	**40; 41**	*
**Punta della Vipera**	**23**	**42.00**	**11.82**	**-**	**-0.9**	**0.4**	**0.4**	**0**	**50**	**Fishtank**	**40; 41**	*
Tiber delta	24	41.84	12.26	R-1198 a	-2.6	1.2	1.2	-3502	132	Peaty clay	42	*
Tiber delta	24	41.84	12.26	R-1198	-2.6	1.2	1.2	-3508	135	Peaty clay	42	*
Tiber delta	24	41.84	12.26	R-888	-8.8	1.2	1.2	-6589	161	Organics	42	*
Tiber delta	24	41.84	12.26	R-1199	-3.6	1.2	1.2	-4099	131	Organics	42	*
Tiber delta	24	41.84	12.26	R-1200	-28.0	1.5	1.5	-8988	215	Organics	42	*
Tiber delta	24	41.84	12.26	R-1200 A/a	-28.0	1.5	1.5	-8980	248	Wood	42	*
Tiber delta	24	41.83	12.26	R-1198a	-2.1	1.2	1.2	-3502	132	Peat	42	*
Tiber delta	24	41.83	12.26	R-1198	-2.1	1.2	1.2	-3508	135	Peat	42	*
Tiber delta	24	41.83	12.26	R-887a	-3.1	1.2	1.2	-3369	265	Wood	42	*
Tiber delta	24	41.83	12.26	R-1623	-3.1	1.2	1.2	-4099	131	Wood	42	*

Site	*Reg.*	*Lat.*	*Long.*	*Lab code*	*RSL (m)*	*Error m (+)*	*Error m (-)*	*Age CE*	*Error*	*Material*	*Reference*	
**Tiber delta**	**24**	**41.83**	**12.26**	**R-1624**	**-2.1**	**1.2**	**1.2**	**-3372**	**259**	**Organics**	**42**	*
**Tiber delta**	**24**	**41.83**	**12.26**	**R-1200**	**-30.0**	**1.2**	**1.2**	**-8988**	**215**	**Silt**	**42**	*
Tiber delta	24	41.83	12.26	R-1200a	-30.0	1.2	1.2	-9103	266	Wood	43	*
Tiber delta	24	41.83	12.27	LTL-1494A	-0.6	0.6	0.6	61	149	Bulk	43	*
Tiber delta	24	41.82	12.28	LYON-8805	-12.3	1.0	1.0	-7692	193	Shell	43	*
Tiber delta	24	41.82	12.28	LYON-8806	-13.8	1.0	1.0	-7620	177	Shell	43	*
Tiber delta	24	41.82	12.28	LYON-8803	-15.3	1.0	1.0	-7969	225	Shell	43	*
Tiber delta	24	41.82	12.28	LYON-8785	-1.5	0.5	0.5	-1460	49	Wood	43	*
**Tiber delta**	**24**	**41.78**	**12.25**	**LY-4198**	**-0.8**	**0.2**	**0.2**	**239**	**162**	**Shell**	**44**	*
**Cala Liberotto**	**25**	**40.44**	**9.78**	**12**	**-33.5**	**1.6**	**1.6**	**-8034**	**305**	**Beachrock**	**45**	*
Posada	25	40.64	9.73	DSH6660	-5.5	1.3	1.3	-4956	119	Organics	46	**
Posada	25	40.64	9.73	DSH6666	-7.2	1.3	1.3	-5335	121	Organics	46	**
**Posada**	**25**	**40.64**	**9.73**	**DSH6668**	**-4.6**	**1.3**	**1.3**	**-5284**	**78**	**Organics**	**46**	**
Posada	25	40.64	9.73	DSH6664	-6.1	1.3	1.3	-5290	72	Organics	46	**
**Posada**	**25**	**40.64**	**9.73**	**DSH6665**	**-7.4**	**1.3**	**1.3**	**-5555**	**70**	**Organics**	**46**	**
**Posada**	**25**	**40.64**	**9.73**	**DSH6667**	**-7.0**	**1.3**	**1.3**	**-5387**	**81**	**Organics**	**46**	**
Posada	25	40.64	9.73	DSH6661	-9.7	1.3	1.3	-5631	95	Organics	46	**
Gulf of Oristano	26	39.90	8.53	DSH6611	-0.8	0.8	0.8	1207	105	Organics	47	**
Gulf of Oristano	26	39.90	8.53	DSH6612	-2.0	0.8	0.8	1091	67	Organics	47	**
Gulf of Oristano	26	39.90	8.53	DSH5650	-4.4	1.3	1.3	-3933	136	Organics	47	**
Gulf of Oristano	26	39.90	8.53	DSH5657	-6.2	1.3	1.3	-5502	116	Organics	47	**
**Gulf of Oristano**	**26**	**39.90**	**8.53**	**DSH5659**	**-8.0**	**1.3**	**1.3**	**-5730**	**121**	**Organics**	**47**	**
Gulf of Oristano	26	39.90	8.53	DSH5658	-4.0	1.3	1.3	-4110	128	Organics	47	**
Gulf of Oristano	26	39.90	8.53	DSH6610	-4.9	0.8	0.8	-3464	159	Organics	47	**
Gulf of Oristano	26	39.90	8.53	DSH5788	-5.9	0.8	0.8	-3513	130	Organics	47	**
**Gulf of Oristano**	**26**	**39.90**	**8.53**	**DSH6541**	**-6.2**	**0.8**	**0.8**	**-3594**	**68**	**Organics**	**47**	**
Gulf of Oristano	26	39.90	8.53	DSH6996_S	-3.6	0.8	0.8	-3697	63	Organics	47	**
**Gulf of Oristano**	**26**	**39.90**	**8.53**	**DSH6997_S**	**-4.4**	**0.8**	**0.8**	**-4115**	**112**	**Organics**	**47**	**
Gulf of Oristano	26	39.89	8.45	AA-92534	-0.3	0.3	1.0	697	81	Organics	48	**
Gulf of Oristano	26	39.89	8.45	AA-92535	-1.4	1.0	1.0	-556	173	Organics	48	**
Gulf of Oristano	26	39.89	8.45	AA-84428	-2.2	1.0	1.0	-2660	189	Organics	48	**
**Gulf of Oristano**	**26**	**39.81**	**8.45**	**14V1**	**-27.0**	**1.1**	**1.1**	**-7454**	**132**	**Shell**	**49**	**
Gulf of Oristano	26	39.74	8.61	P26-4	-1.7	1.1	1.1	-245	114	Shell	50	**
Gulf of Oristano	26	39.74	8.61	P26-3.45	-1.5	1.1	1.1	-30	158	Shell	50	**
**Sant'Antioco**	**27**	**39.07**	**8.46**	**-**	**-1.3**	**0.5**	**0.5**	**-300**	**100**	**Archeol. structure**	**51**	**

Site	*Reg.*	*Lat.*	*Long.*	*Lab code*	*RSL (m)*	*Error m (+)*	*Error m (-)*	*Age CE*	*Error*	*Material*	*Reference*	
**Sant'Antioco**	**27**	**39.05**	**8.47**	**-**	**-1.1**	**0.3**	**0.3**	**60**	**20**	**Ceramics in beachrocks**	**51**	**
Malfatano	28	38.90	8.80	LTL-8291A	-0.2	0.2	1.0	134	165	Shell	52	**
Malfatano	28	38.90	8.80	LTL-8292A	-1.3	1.0	1.0	-89	177	Shell	52	**
Malfatano	28	38.90	8.80	LTL-8293A	-2.3	1.0	1.0	-1104	192	Shell	52	**
**Cagliari plain**	**29**	**39.21**	**9.09**	**GX-29077**	**-29.5**	**1.0**	**1.0**	**-7729**	**170**	**Shell**	**53**	**
**Cagliari plain**	**29**	**39.21**	**9.09**	**GX-25487**	**-45.5**	**1.6**	**1.6**	**-8891**	**251**	**Shell**	**53**	**
**Sarinola**	**30**	**41.25**	**13.60**	**-**	**-0.9**	**0.4**	**0.4**	**0**	**50**	**Fishtanks**	**40;41**	*
Volturno	30	40.97	14.00	CV-10	-2.5	0.6	0.6	-2579	285	Wood	54	*
Volturno	30	40.97	14.00	CV-5	-7.5	0.6	0.6	-5567	96	Wood	54	*
Volturno	30	40.94	14.03	G1bis 5/41	-1.0	0.5	0.5	-163	198	Shell	55	*
Salerno Bay	31	40.42	15.00	ROME-665	-14.7	1.1	1.1	-7305	221	Organics	56	*
Salerno Bay	31	40.42	15.00	ROME-666	-16.3	1.1	1.1	-7379	192	Wood debris	56	*
Salerno Bay	31	40.39	15.00	-	-3.2	1.1	1.1	-5269	262	Foraminifera	57	*
Salerno Bay	31	40.39	15.00	-	-0.5	1.1	1.1	-2150	50	Tephra layer	57	*
**Salerno Bay**	**31**	**40.39**	**15.00**	**-**	**-9.8**	**1.1**	**1.1**	**-6253**	**196**	**Shell**	**57**	*
**Salerno Bay**	**31**	**40.39**	**15.00**	**-**	**-8.3**	**1.1**	**1.1**	**-6442**	**193**	**Shell**	**57**	*
Salerno Bay	31	40.49	14.94	Rome-815	-1.5	0.7	0.7	507	117	Organics	58	
Salerno Bay	31	40.49	14.94	Rome-811	-4.5	0.7	0.7	-2081	194	Organics	58	
**G. of Gaeta-Volturno**	**31**	**40.49**	**14.94**	**Rome-816**	**-7.5**	**0.0**	**1.1**	**-7371**	**195**	**Organics**	**58**	
**SanVito-C.Gallo**	**32**	**38.21**	**13.28**	**R-2580**	**-0.4**	**0.3**	**0.3**	**1583**	**111**	**Vermetid**	**59**	*
**SanVito-C.Gallo**	**32**	**38.11**	**12.71**	**R-2764**	**-0.3**	**0.3**	**0.3**	**1830**	**121**	**Vermetid**	**59**	*
**SanVito-C.Gallo**	**32**	**38.17**	**12.71**	**R-2741**	**-0.3**	**0.3**	**0.3**	**1835**	**115**	**Vermetid**	**59**	*
**SanVito-C.Gallo**	**32**	**38.17**	**12.71**	**R-2742**	**-0.3**	**0.3**	**0.3**	**1835**	**115**	**Vermetid**	**59**	*
**MarsalaSound**	**33**	**37.89**	**12.45**	**OZE-613**	**-1.1**	**0.6**	**0.6**	**-851**	**140**	**Shell**	**60**	*
**MarsalaSound**	**33**	**37.89**	**12.45**	**OZE-612**	**-0.4**	**0.4**	**0.6**	**-549**	**175**	**Shell**	**60**	*
**MarsalaSound**	**33**	**37.87**	**12.48**	**OZE-611**	**-0.2**	**0.2**	**0.6**	**1199**	**139**	**Shell**	**60**	*
**MarsalaSound**	**33**	**37.84**	**12.45**	**OZE-610**	**-0.1**	**0.1**	**0.6**	**1672**	**156**	**Shell**	**60**	*
**MarsalaSound**	**33**	**37.87**	**12.45**	**OZE-609**	**-0.1**	**0.1**	**0.6**	**903**	**141**	**Shell**	**60**	*
Vendicari-Pachino	34	36.80	15.09	POZ-17907	-4.4	1.1	1.1	-2696	192	Shell	61	*

Site	*Reg.*	*Lat.*	*Long.*	*Lab code*	*RSL (m)*	*Error m (+)*	*Error m (-)*	*Age CE*	*Error*	*Material*	*Reference*	
Vendicari-Pachino	34	36.80	15.09	POZ-17906	-5.9	1.1	1.1	-3097	202	Shell	61	*
Vendicari-Pachino	34	36.70	15.11	LTL4282A	0.0	0.0	1.1	528	153	Shell	62	*
Vendicari-Pachino	34	36.70	15.11	LTL4284A	0.0	0.0	1.1	579	159	Shell	62	*
Vendicari-Pachino	34	36.70	15.11	LTL4285A	-2.1	1.1	1.1	-1992	210	Shell	62	*
Vendicari-Pachino	34	36.70	15.10	LTL4887A	-0.4	0.4	1.1	423	188	Shell	62	*
Vendicari-Pachino	34	36.70	15.10	LTL4888A	-3.2	1.1	1.1	-2874	234	Shell	62	*
Vendicari-Pachino	34	36.70	15.10	LTL4889A	0.0	0.0	1.1	1829	121	Shell	62	*
Vendicari-Pachino	34	36.70	15.10	LTL4903A	-1.8	1.1	1.1	-935	163	Shell	62	*
Malta	35	35.93	14.41	POZ-42441	-7.0	1.0	1.0	-4211	136	Charcoal	63	*
Malta	35	35.93	14.41	SACA-11668	-9.2	0.7	0.7	-5077	129	Peat	63	*
Malta	35	35.93	14.41	SACA-11669	-9.6	0.5	0.5	-5449	73	Charcoal	63	*
Malta	35	35.93	14.41	POZ-42439	-11.1	1.0	1.0	-5571	88	Charcoal	63	*
Malta	35	35.93	14.41	POZ-42444	-8.2	1.0	1.0	-4946	99	Charcoal	63	*
**Malta**	**35**	**35.84**	**14.89**	**LTL14449A3**	**-14.8**	**0.5**	**0.5**	**-5677**	**56**	**Speleothem**	**64**	*
ElGuettate-Dreiaa	36	34.2	10.03	-	0.5	1.1	1.1	-3511	175	Shell	65	*
ElGuettate-Dreiaa	36	34.2	10.03	-	-0.6	0.9	0.9	-5502	257	Shell	65	*
ElGuettate-Dreiaa	36	34.2	10.03	-	0.2	0.7	0.7	-3467	182	Shell	65	*
ElGuettate-Dreiaa	36	34.17	10.02	SACA-12307	0.4	0.7	0.7	1304	131	Shell	66	*
ElGuettate-Dreiaa	36	34.17	10.02	SACA-12306	0.7	0.7	0.7	1192	144	Shell	66	*
ElGuettate-Dreiaa	36	34.16	10.01	BETA-282579	0.1	1.1	1.1	-4721	201	Shell	66	*
**Humt**	**37**	**33.73**	**10.72**	**POZ-2590**	**0.4**	**0.8**	**0.8**	**-2344**	**211**	**Shell**	**67**	*
**ElBibane-Boujmel**	**38**	**33.3**	**11.12**	**LU-2656**	**-0.3**	**0.8**	**0.8**	**-1343**	**224**	**Beachrock cement**	**68**	*
**ElBibane-Boujmel**	**38**	**33.3**	**11.12**	**LU-2653**	**-0.4**	**0.8**	**0.8**	**-1185**	**225**	**Beachrock cement**	**68**	*

Site	Reg.	Lat.	Long.	Lab code	RSL (m)	Error m (+)	Error m (-)	Age CE	Error	Material	Reference	
**ElBibane-Boujmel**	**38**	**33.3**	**11.12**	**LU-2652**	**0.0**	**0.8**	**0.8**	**-2550**	**273**	**Beachrock cement**	**68**	*
ElBibane-Boujmel	38	33.29	11.09	na	0.4	0.6	0.6	155	255	Bulk material	69	*
ElBibane-Boujmel	38	33.29	11.09	na	0.2	0.6	0.6	-567	624	Bulk material	69	*
ElBibane-Boujmel	38	33.29	11.09	na	0.0	0.6	0.6	-1208	291	Bulk material	69	*
ElBibane-Boujmel	38	33.29	11.09	na	-0.1	0.6	0.6	-2160	462	Bulk material	69	*
ElBibane-Boujmel	38	33.29	11.09	na	-0.1	0.6	0.6	-2362	466	Bulk material	69	*
**ElBibane-Boujmel**	**38**	**33.29**	**11.09**	**na**	**-0.3**	**0.6**	**0.6**	**-4878**	**415**	**Bulk material**	**69**	*
Venice lagoon	39	45.4	12.25	ROME-1205	-1.1	0.5	0.5	1359	66	Bulk material	70	*
Venice lagoon	39	45.35	12.32	OZG-805	-0.7	0.6	0.6	1168	110	Bulk material	70	*
Venice lagoon	39	45.47	12.41	OxA-10717	-1.6	1.2	1.2	1138	102	Plant remains	71	*
Venice lagoon	39	45.48	12.33	OZ-G311	-0.9	0.6	0.6	1021	126	Shell	70	*
Venice lagoon	39	45.35	12.32	OZG-332	-0.4	0.4	1.0	689	199	Shell	70	*
Venice lagoon	39	45.47	12.41	OxA-10722	-0.7	0.7	1.2	487	84	Plant remains	71	*
**Venice lagoon**	**39**	**45.49**	**12.35**	**OZ-G317**	**-0.3**	**0.3**	**1.1**	**419**	**211**	**Shell**	**70**	*
Venice lagoon	39	45.36	12.25	OZG-320	-1.1	0.6	0.6	271	212	Shell	70	*
Venice lagoon	39	45.47	12.41	OxA-6784	-0.6	0.6	0.7	254	127	Wood	72	*
Venice lagoon	39	45.47	12.41	OZF-487	-1.4	1.2	1.2	210	126	Plant remains	71	*
Venice lagoon	39	45.35	12.32	OZG-333	-0.7	0.7	1.0	144	217	Shell	70	*
Venice lagoon	39	45.47	12.41	OxA-8629	-0.9	0.7	0.7	126	100	Leaf	72	*
Venice lagoon	39	45.47	12.41	OZ-E696	-1.0	0.7	0.7	-97	198	Foraminifera	72	*
Venice lagoon	39	45.47	12.29	LTL-1631A	-1.4	0.6	0.6	-553	191	Peat	73	*
**Venice lagoon**	**39**	**45.35**	**12.32**	**OZG-322**	**-1.4**	**0.7**	**0.7**	**-634**	**233**	**Foraminifera**	**70**	*
Venice lagoon	39	45.54	12.44	CARG-12	-0.9	0.9	1.2	-1143	122	-	74	*
Venice lagoon	39	45.47	12.41	OZ-E697	-2.2	0.7	0.7	-1611	187	Shell	72	*
Venice lagoon	39	45.47	12.41	OZ-E698	-2.2	0.7	0.7	-1685	188	Foraminifera	72	*
Venice lagoon	39	45.47	12.41	OZF-484	-2.0	1.2	1.2	-2125	342	Plant remains	71	*
Venice lagoon	39	45.47	12.41	OZ-E699	-1.8	0.7	0.7	-2239	207	Foraminifera	72	*
Venice lagoon	39	45.47	12.41	OxA-1076	-1.0	1.0	1.2	-2300	157	Plant remains	71	*
**Venice lagoon**	**39**	**45.48**	**12.41**	**GX-26939**	**-4.3**	**0.8**	**0.8**	**-2723**	**147**	**Peat**	**71**	*
**Venice lagoon**	**39**	**45.43**	**12.45**	**OZF-080**	**-3.0**	**0.6**	**0.6**	**-3606**	**237**	**Shell**	**75**	*

Site	*Reg.*	*Lat.*	*Long.*	*Lab code*	*RSL (m)*	*Error m (+)*	*Error m (-)*	*Age CE*	*Error*	*Material*	*Reference*	
**Friuli lagoons**	**40**	**45.67**	**12.93**	**B6 Paris Sud**	**-5.5**	**0.7**	**0.7**	**-4550**	**386**	**Organics**	**76**	*
**Friuli lagoons**	**40**	**45.64**	**12.93**	**Ua-24876**	**-4.7**	**0.7**	**0.7**	**-4577**	**75**	**Peat**	**77**	*
**Friuli lagoons**	**40**	**45.63**	**12.88**	**Beta-184251**	**-9.0**	**0.8**	**0.8**	**-5006**	**209**	**Peat**	**77**	*
**Friuli lagoons**	**40**	**45.62**	**12.75**	**Ua-24049**	**-9.3**	**0.8**	**0.8**	**-5547**	**75**	**Peat**	**78**	*
Friuli lagoons	40	45.62	12.95	ParisS-4209	-0.6	0.6	0.6	329	320	Shell	76	*
**Friuli lagoons**	**40**	**45.6**	**12.64**	**Beta-168127**	**-6.5**	**0.9**	**0.9**	**-5462**	**77**	**Peat**	**79**	*
**Friuli lagoons**	**40**	**45.58**	**12.47**	**Beta-157974**	**-3.4**	**0.6**	**0.6**	**-1398**	**101**	**Peat**	**79**	*
**Friuli lagoons**	**40**	**45.57**	**12.44**	**-**	**-1.1**	**0.7**	**0.7**	**50**	**50**	**Archeological structure**	**79**	*
Friuli lagoons	40	45.65	12.64	Beta-170844	-1.9	0.8	0.8	-1933	199	Peat Organics	79	*
Gulf of Trieste	41	45.77	13.57	DSH-869	-1.0	1.0	1.1	13	53	Wood	80	*
Gulf of Trieste	41	45.77	13.57	DSH-815	-0.7	0.7	1.0	1086	115	Shell	80	*
Gulf of Trieste	41	45.77	13.57	Anto 2	0.0	0.0	1.0	1401	79	Shell	80	*
Gulf of Trieste	41	45.77	13.57	Anto 4	0.0	0.0	1.0	1017	151	Shell	80	*
Gulf of Trieste	41	45.67	13.67	GT1-200	-25.2	1.2	1.2	-8369	93	Organics	81	*
Gulf of Trieste	41	45.64	13.76	POZ-15854	-0.1	0.1	0.8	208	125	Shell	82	*
Gulf of Trieste	41	45.64	13.76	POZ-15856	-0.1	0.1	0.8	-1234	177	Shell	82	*
Gulf of Trieste	41	45.607	13.52	Core GT3-64	-25.2	1.1	1.1	-7627	125	Shell	81	*
Gulf of Trieste	41	45.54	13.72	na	-0.2	0.2	1.2	1037	190	Shell	83	*
Istria	42	45.52	13.52	Core-V6	-25.0	1.2	1.2	-7952	319	Shell	83	*
**Istria**	**42**	**45.28**	**13.6**	**POZ-15849**	**-0.2**	**0.2**	**1.0**	**904**	**114**	**Shell**	**84**	*
**Istria**	**42**	**45.28**	**13.6**	**POZ-15850**	**-1.3**	**1.0**	**1.0**	**-1054**	**141**	**Shell**	**84**	*
Istria	42	45.11	13.62	POZ-15845	-0.1	0.1	1.0	1329	83	Shell	84	*
**Istria**	**42**	**45.11**	**13.62**	**POZ-15846**	**-2.9**	**1.0**	**1.0**	**-3063**	**177**	**Shell**	**84**	*
**Istria**	**42**	**45.11**	**13.62**	**POZ-15847**	**-2.9**	**1.0**	**1.0**	**-3051**	**170**	**Shell**	**84**	*
Romagna shelf	43	44.84	12.13	BETA-187	-20.2	0.9	0.9	-7257	188	Organics	85	*
Romagna shelf	43	44.72	12.02	na	-24.6	0.9	0.9	-8815	325	Peat	86	*
Romagna shelf	43	44.63	12.07	na	-23.4	0.8	0.8	-8088	333	Organics	86	*
Romagna shelf	43	44.49	12.89	na	-41.8	1.0	1.0	-10115	311	Peat	86	*
Romagna shelf	43	44.49	12.89	na	-41.7	1.0	1.0	-10159	281	Peat	86	*
Romagna shelf	43	44.88	12.10	ROMA-187Mezz2	-4.1	0.6	0.6	-3102	221	Organic sediment	85	*
Romagna shelf	43	44.30	12.25	ENEA-Bolog-	-7.6	0.8	0.8	-4084	260	Organic clay	85	*
Romagna shelf	43	44.23	12.36	LLNL-Livermore	-8.2	1.4	1.4	-4687	136	Organic clay	85	*

Site	*Reg.*	*Lat.*	*Long.*	*Lab code*	*RSL (m)*	*Error m (+)*	*Error m (-)*	*Age CE*	*Error*	*Material*	*Reference*	
Romagna shelf	43	44.60	11.92	ENEA-Bolog-	-8.3	1.5	1.5	-5168	311	Organic clay	85	*
Romagna shelf	43	44.30	12.25	ENEA-Bolog-	-10.6	0.8	0.8	-5684	193	Organic clay	85	*
**Romagna shelf**	**43**	**44.62**	**12.01**	**ETH**	**-10.8**	**1.2**	**1.2**	**-5953**	**367**	**Shell**	**87**	*
North Dalmatia	44	44.54	14.91	LYON-9044	0.0	0.0	0.2	1258	34	Charcoal	88	*
North Dalmatia	44	44.54	14.91	LYON-9045	0.0	0.0	0.7	1069	82	Plant remains	88	*
North Dalmatia	44	44.54	14.91	LYON-9048	0.0	0.0	0.6	955	62	Plant remains	88	*
North Dalmatia	44	44.54	14.91	LYON-9049	0.0	0.0	0.6	1098	84	Plant remains	88	*
**Vis-Bisevo**	**45**	**43.08**	**16.18**	**Z-4301**	**-0.3**	**0.3**	**0.3**	**1348**	**70**	***L. byssoides***	**89**	*
**Vis-Bisevo**	**45**	**43.08**	**16.18**	**Z-4302**	**-0.3**	**0.3**	**0.3**	**1374**	**64**	***L. byssoides***	**89**	*
**Vis-Bisevo**	**45**	**43.08**	**16.18**	**Z-4303**	**-0.3**	**0.3**	**0.3**	**1707**	**244**	***L. byssoides***	**89**	*
**Vis-Bisevo**	**45**	**43.08**	**16.18**	**Z-4848**	**-0.3**	**0.3**	**0.3**	**1548**	**95**	***L. byssoides***	**89**	*
**Vis-Bisevo**	**45**	**43.08**	**16.18**	**Z-4849**	**-0.3**	**0.3**	**0.3**	**1336**	**74**	***L. byssoides***	**89**	*
**Vis-Bisevo**	**45**	**43.08**	**16.18**	**Z-4850**	**-0.3**	**0.3**	**0.3**	**1532**	**100**	***L. byssoides***	**89**	*
**Vis-Bisevo**	**45**	**43.08**	**16.18**	**Z-4851**	**-0.3**	**0.3**	**0.3**	**1550**	**90**	***L. byssoides***	**89**	*
**Vis-Bisevo**	**45**	**43.08**	**16.18**	**Z-4852**	**-0.3**	**0.3**	**0.3**	**1640**	**156**	***L. byssoides***	**89**	*
**Vis-Bisevo**	**45**	**43.08**	**16.18**	**Z-4307**	**-0.7**	**0.3**	**0.3**	**783**	**187**	***L. byssoides***	**89**	*
**Vis-Bisevo**	**45**	**43.08**	**16.18**	**Z-4308**	**-0.7**	**0.3**	**0.3**	**776**	**105**	***L. byssoides***	**89**	*
**Vis-Bisevo**	**45**	**43.08**	**16.18**	**Z-4309**	**-0.7**	**0.3**	**0.3**	**548**	**115**	***L. byssoides***	**89**	*
**Vis-Bisevo**	**45**	**43.08**	**16.18**	**Z-4310**	**-0.7**	**0.3**	**0.3**	**546**	**110**	***L. byssoides***	**89**	*
**Vis-Bisevo**	**45**	**43.08**	**16.18**	**Z-4311**	**-0.7**	**0.3**	**0.3**	**533**	**142**	***L. byssoides***	**89**	*
**Vis-Bisevo**	**45**	**43.08**	**16.18**	**Z-4312**	**-0.7**	**0.3**	**0.3**	**722**	**51**	***L. byssoides***	**89**	*
**Vis-Bisevo**	**45**	**43.08**	**16.18**	**Z-4642**	**-0.7**	**0.3**	**0.3**	**713**	**55**	***L. byssoides***	**89**	*
**Vis-Bisevo**	**45**	**42.98**	**16.01**	**Z-4693**	**-0.2**	**0.2**	**0.3**	**1342**	**64**	***L. byssoides***	**89**	*
**Vis-Bisevo**	**45**	**42.98**	**16.01**	**Z-4694**	**-0.2**	**0.2**	**0.3**	**1542**	**98**	***L. byssoides***	**89**	*
**Vis-Bisevo**	**45**	**42.98**	**16.01**	**Z-4685**	**-0.7**	**0.3**	**0.3**	**554**	**121**	***L. byssoides***	**89**	*
**Vis-Bisevo**	**45**	**42.98**	**16.01**	**Z-4686**	**-0.7**	**0.3**	**0.3**	**802**	**142**	***L. byssoides***	**89**	*
**Vis-Bisevo**	**45**	**42.98**	**16.01**	**Z-4692**	**-1.5**	**0.3**	**0.3**	**-715**	**176**	***L. byssoides***	**89**	*
**Vis-Bisevo**	**45**	**43.01**	**16.22**	**Z-4695**	**-0.2**	**0.2**	**0.3**	**1384**	**79**	***L. byssoides***	**89**	*
**Vis-Bisevo**	**45**	**43.01**	**16.22**	**Z-4696**	**-0.2**	**0.2**	**0.3**	**1372**	**72**	***L. byssoides***	**89**	*
Battaglia lake	46	41.90	16.13	GX-30414	-3.7	1.1	1.1	-4014	211	Charcoal	90	*
Battaglia lake	46	41.90	16.13	LTL-664A	-5.6	0.6	0.6	-4860	132	Seed	90	*
Battaglia lake	46	41.90	16.13	LTL-456A	-6.1	0.6	0.6	-4999	206	Seed	90	*
Frattarolo lagoon	47	41.57	15.84	GX-24886	-1.0	1.0	1.0	-1334	102	Plant remains	90	*
Frattarolo lagoon	47	41.57	15.84	GX-30408	-1.6	0.3	0.3	-1352	136	Sediment	91	*

Site	Reg.	Lat.	Long.	Lab code	RSL (m)	Error m (+)	Error m (-)	Age CE	Error	Material	Reference	
Frattarolo lagoon	47	41.57	15.84	UA-15675	-1.1	1.0	1.0	-1448	168	Plant remains	92	*
Frattarolo lagoon	47	41.57	15.84	UA-14598	-1.2	1.0	1.0	-1460	196	Plant remains	92	*
Frattarolo lagoon	47	41.57	15.84	LTL-2078A	-2.2	1.0	1.0	-2467	160	Sediment	91	*
Frattarolo lagoon	47	41.57	15.84	GX-28575	-2.5	1.0	1.0	-3146	203	Shell	91	*
Alimini lake	48	40.17	18.44	UA-13189	-1.6	1.0	1.0	920	229	Organics	93	*
Alimini lake	48	40.17	18.44	UA-12577	-2.2	0.7	0.7	-25	148	Organics	93	*
Alimini lake	48	40.17	18.44	UA-12578	-3.4	0.5	0.5	-2120	174	Organics	93	*
Alimini lake	48	40.17	18.44	UA-12054	-4.0	0.5	0.5	-3512	144	Organics	93	*

Supplementary table 2. Rates of Med-SL ± 1σ at 0.1 ka intervals.

Year CE	*mm a-1*	*1s*	*P>0*	*Year CE*	*mm a-1*	*1s*	*P>0*
-7950	8.75	0.9	1.0	-2950	1.00	0.7	0.9
-7850	8.60	0.9	1.0	-2850	1.00	0.7	0.9
-7750	8.50	0.9	1.0	-2750	1.00	0.7	0.9
-7650	8.35	0.9	1.0	-2650	0.95	0.7	0.9
-7550	8.25	0.9	1.0	-2550	0.85	0.7	0.9
-7450	8.10	0.9	1.0	-2450	0.80	0.7	0.9
-7350	7.95	0.9	1.0	-2350	0.75	0.7	0.8
-7250	7.80	0.9	1.0	-2250	0.65	0.7	0.8
-7150	7.70	0.9	1.0	-2150	0.50	0.7	0.8
-7050	7.60	0.8	1.0	-2050	0.45	0.7	0.7
-6950	7.50	0.8	1.0	-1950	0.45	0.7	0.7
-6850	7.40	0.8	1.0	-1850	0.45	0.7	0.7
-6750	7.35	0.8	1.0	-1750	0.50	0.7	0.7
-6650	7.20	0.8	1.0	-1650	0.50	0.7	0.8
-6550	7.00	0.8	1.0	-1550	0.50	0.7	0.8
-6450	6.80	0.8	1.0	-1450	0.55	0.7	0.8
-6350	6.55	0.8	1.0	-1350	0.55	0.7	0.8
-6250	6.30	0.8	1.0	-1250	0.55	0.7	0.8
-6150	6.00	0.8	1.0	-1150	0.55	0.7	0.8
-6050	5.75	0.8	1.0	-1050	0.55	0.7	0.8
-5950	5.45	0.8	1.0	-950	0.50	0.7	0.8
-5850	5.25	0.8	1.0	-850	0.50	0.7	0.8
-5750	5.05	0.8	1.0	-750	0.50	0.7	0.8
-5650	4.85	0.8	1.0	-650	0.50	0.7	0.8
-5550	4.60	0.8	1.0	-550	0.50	0.7	0.8
-5450	4.25	0.8	1.0	-450	0.45	0.7	0.7
-5350	3.90	0.8	1.0	-350	0.40	0.7	0.7
-5250	3.55	0.8	1.0	-250	0.40	0.7	0.7
-5150	3.30	0.8	1.0	-150	0.40	0.7	0.7
-5050	3.10	0.8	1.0	-50	0.40	0.7	0.7
-4950	2.95	0.8	1.0	50	0.40	0.7	0.7
-4850	2.75	0.8	1.0	150	0.40	0.7	0.7
-4750	2.60	0.8	1.0	250	0.40	0.7	0.7
-4650	2.40	0.8	1.0	350	0.40	0.7	0.7
-4550	2.25	0.8	1.0	450	0.35	0.7	0.7
-4450	2.10	0.8	1.0	550	0.30	0.7	0.7
-4350	1.95	0.8	1.0	650	0.30	0.7	0.7
-4250	1.85	0.8	1.0	750	0.30	0.7	0.7
-4150	1.75	0.8	1.0	850	0.30	0.7	0.7
-4050	1.65	0.8	1.0	950	0.40	0.7	0.7
-3950	1.50	0.8	1.0	1050	0.45	0.7	0.7

Year CE	mm a-1	1s	P>0	Year CE	mm a-1	1s	P>0
-3850	1.35	0.8	1.0	1150	0.45	0.7	0.7
-3750	1.25	0.8	1.0	1250	0.45	0.7	0.7
-3650	1.15	0.8	0.9	1350	0.45	0.7	0.7
-3550	1.10	0.8	0.9	1450	0.45	0.7	0.8
-3450	1.05	0.8	0.9	1550	0.50	0.7	0.8
-3350	1.05	0.8	0.9	1650	0.50	0.7	0.8
-3250	1.00	0.8	0.9	1750	0.65	0.6	0.8
-3150	1.00	0.8	0.9	1850	0.75	0.6	0.9
-3050	1.00	0.8	0.9	1950	1.05	0.6	1.0

Supplementary References

1] Vacchi, M., et al..New relative sea-level insights into the isostatic history of the Western Mediterranean. *Quaternary Science Reviews* **201**, 396-408 (2018).

2] Galassi, G., & Spada, G. Sea-level rise in the Mediterranean Sea by 2050: Roles of terrestrial ice melt, steric effects and glacial isostatic adjustment. *Global and Planetary Change* **123**, 55-66 (2014)

3] Peltier, W.R. Global glacial isostasy and the surface of the ice-age Earth: the ICE-5G (VM2) model and GRACE. *Annual Review of Earth and Planetary Sciences* **32**, 111-149 (2004).

4] Lambeck, K., Purcell, A. Sea-level change in the Mediterranean Sea since the LGM: model predictions for tectonically stable areas. *Quaternary Science Reviews* **24(18-19)**, 1969-1988 (2005).

5] Vacchi, M., et al. Multiproxy assessment of Holocene relative sea-level changes in the western Mediterranean: Sea-level variability and improvements in the definition of the isostatic signal. *Earth-Science Reviews* **155**, 172-197 (2016).

6] Ferrer-García, C., Blázquez-Morilla, A.M. The evolution of the Albufereta lagoon (western Mediterranean): climate cycles and sea-level changes. *Journal of Coastal Research* **28(6),** 1617-1626 (2012).

7] Brisset, E., Burjachs, F., Navarro, B. J. B., de Pablo, J. F. L. Socio-ecological adaptation to Early-Holocene sea-level rise in the western Mediterranean. *Global and planetary change* **169,** 156-167 (2018).

8] Dupré, M., Fumanal, M. P., Sanjaume, E., Santisteban, C., Usera. J. Quaternary evolution of Pego coastal lagoon (Southern Valencia. Spain). *Palaeogeography. Palaeoclimatology. Palaeoecology* **68**, 291-299 (1988).

9] Ruiz, J. M., Carmona, P. La llanura deltaica de los ríos Júcar y Turia y la Albufera de Valencia. *Geomorfologia i Quaternari litoral. Homenatge al Dr. V. Rosselló* **1**, 399-419 (2005).

10] Marco-Barba, J., Holmes, J. A., Mesquita-Joanes, F., Miracle, M. R. The influence of climate and sea-level change on the Holocene evolution of a Mediterranean coastal lagoon: Evidence from ostracod palaeoecology and geochemistry. *Geobios* **46(5)**. 409-421 (2013).

11] Sanjaume, E., Segura, F., Garcia, M. L., Pardo, J. Recent sedimentation in Valencia Lagoon: preliminary results. *Journal of Coastal Research* **8 (3),** 688-698 (1992).

12] Carmona, P., Ballester, J.P. Geomorphology, geoarchaeology and ancient settlement in the Valencian Gulf (Spain). *Méditerranée. Revue géographique des pays méditerranéens/Journal of Mediterranean geography* **117**, 61-72 (2011).

13] Rodríguez-Pérez, A., Blázquez, A. M., Guillem, J., Usera, J. Maximum flood area during MIS 1 in the Almenara marshland (western Mediterranean): Benthic foraminifera and sedimentary record. *The Holocene* **28(9)**, 1452-1466 (2018).

14] Carmona, P., et al. Environmental evolution and mid–late Holocene climate events in the Valencia lagoon (Mediterranean coast of Spain). *The Holocene* **26(11)**. 1750-1765 (2016).

15] Blázquez, A. M., Rodríguez-Pérez, A., Torres, T., Ortiz. J. E. Evidence for Holocene sea level and climate change from Almenara marsh (western Mediterranean). *Quaternary Research* **88(2),** 206-222 (2017).

16] Giaime M., et al. In search of Pollentia's southern harbour: Geoarchaeological evidence from the Bay of Alcúdia (Mallorca. Spain). *Palaeogeography, Palaeoclimatology, Palaeoecology* **466**, 184-201 (2017).

17] Yll, R., Pantaleón-Cano, J., Pérez-Obiol, R.P., Roure, J. M. Cambio climático y transformación del medio durante el Holoceno en las Islas Baleares. Congrés del Neolític a la Península Ibérica. *Saguntum Extra* **2**, 45-51 (1999).

18] Burjachs, F., Pérez-Obiol, R., Roure, J. M., Julià, R. Dinámica de la vegetación durante el Holoceno en la isla de Mallorca. *Trabajos de Palinología básica y aplicada* 199-210 (1994).

19] Cearreta, A., Benito, X., Ibáñez, C., Trobajo, R., Giosan, L. Holocene palaeoenvironmental evolution of the Ebro Delta (Western Mediterranean Sea): Evidence for an early construction based on the benthic foraminiferal record. *The Holocene* **26(9),** 1438-1456 (2016).

20] Riera-Mora, S. Esteban-Amat, A. Vegetation history and human activity during the last 6000 years on the central Catalan coast (northeastern Iberian Peninsula). *Vegetation History and Archaeobotany* **3(1).** 7-23 (1994).

21] Daura, J, et al. Palaeoenvironmental record of the Cal Maurici wetland sediment archive in Barcelona (NE Iberian Peninsula) between c. 6000 and 4000 cal. yr BP. *The Holocene* **26(7)**. 1020-1039 (2016).

22] Ejarque, A. et al. Coastal evolution in a Mediterranean microtidal zone: Mid to Late Holocene natural dynamics and human management of the Castelló lagoon. NE Spain. *PloS one* **11(5)**. e0155446, (2016).

23] Laborel, J., et al. Biological evidence of sea-level rise during the last 4500 years on the rocky coasts of continental southwestern France and Corsica. *Marine Geology* **120(3)**, 203-223 (1994).

24] Sabatier, P. et al., Holocene evolution of a Languedocian lagoonal environment controlled by inherited coastal morphology (South of France). *Bulletin de la Societe Geologique de France* **181(1)**. 27-36 (2010).

25]Vella, C., Provansal, M. Relative sea-level rise and neotectonic events during the last 6500yr on the southern eastern Rhône delta. France. *Marine Geology* **170(1)** 27-39 (2000).

26] Amorosi, A. Rossi, V. Vella, C. Stepwise post-glacial transgression in the Rhône Delta area as revealed by high-resolution core data. *Palaeogeography, Palaeoclimatology, Palaeoecology* **374**, 314-326 (2013).

27] Morhange, C., Laborel, J., Hesnard, A. Changes of relative sea level during the past 5000 years in the ancient habour of Marseilles. Southern France. *Palaeogeography, Palaeoclimatology, Palaeoecology* **166(3)**, 319-329 (2001).

28] Vella, C., et al. M. Evolution of the Rhône delta plain in the Holocene. *Marine Geology* **222**, 235-265 (2005).

29] Allinne, et al. Archéologie et paléoenvironnement sur le site du pont romain des Esclapes (Fréjus. Var). *ArcheoSciences. Revue d'archéométrie* **(30)**, 181-196 (2006).

30] Devillers, B., Excoffon, P., Morhange, C., Bonnet, S., Bertoncello, F. Relative sea-level changes and coastal evolution at Forum Julii (Fréjus. Provence). *Comptes Rendus Geoscience* **339(5)**. 329-336 (2007).

31] Morhange, C. et al. Relative Sea-Level Changes During Roman Times in the Northwest Mediterranean: The 1st Century AD Fish Tank of Forum Julii. Fréjus. France. *Geoarchaeology* **28(4),** 363-372 (2013).

32] Colombaroli, D., Marchetto, A., Tinner, W. Long-term interactions between Mediterranean climate. vegetation and fire regime at Lago di Massaciuccoli (Tuscany. Italy). *Journal of Ecology* **95(4)**, 755-770 (2007).

33] Carboni. M. G., et al. Palaeoenvironmental reconstruction of late Quaternary foraminifera and molluscs from the ENEA borehole (Versilian plain. Tuscany. Italy). *Quaternary Research* **74(2)**, 265-276 (2010).

34] Kaniewski. D., et al. Holocene evolution of Portus Pisanus. the lost harbour of Pisa. Scientific reports **8(1)**, 11625 (2018).

35] Ghilardi, M., et al. Reconstructing the landscape evolution and the human occupation of the Lower Sagone River (Western Corsica. France) from the Bronze Age to the Medieval period. *Journal of Archaeological Science: Reports* **12**, 741-754 (2017).

36] Ghilardi, M., et al., Enregistrements d'événements extrêmes et évolution des paysages dans les basses vallées fluviales du Taravo et du Sagone (Corse occidentale. France) au cours de l'âge du Bronze moyen à final: une perspective géoarchéologique. *Géomorphologie: relief. processus. Environnement* **23(1)**, 15-35 (2017).

37] Vacchi, M., Ghilardi, M., Spada, G., Currás, A., Robresco, S. New insights into the sea-level evolution in Corsica (NW Mediterranean) since the late Neolithic. *Journal of Archaeological Science: Reports* **12**, 782-793 (2017).

38] Poher, Y., Ponel, P., Médail, F., Andrieu-Ponel, V., Guiter, F. Holocene environmental history of a small Mediterranean island in response to sea-level changes. climate and human impact. *Palaeogeography, Palaeoclimatology, Palaeoecology*, **465,** 247-263 (2017).

39] Lambeck, K., Anzidei, M., Antonioli, F., Benini, A., Verrubbi, V. Tyrrhenian sea level at 2000 BP: evidence from Roman age fish tanks and their geological calibration. *Rendiconti Lincei. Scienze Fisiche e Naturali,* **29(1)**, 69-80 (2018).

40] Lambeck, K., Anzidei, M., Antonioli, F., Benini, A., Esposito. A. Sea level in Roman time in the Central Mediterranean and implications for recent change. *Earth and Planetary Science Letters* **224(3)**, 563-575 (2004).

41] Evelpidou, N., et al. Late Holocene sea level reconstructions based on observations of Roman fish tanks. Tyrrhenian Coast of Italy. *Geoarchaeolog*y **27(3)**, 259-277 (2012).

42] Marra, F., Bozzano, F., Cinti. F. R. Chronostratigraphic and lithologic features of the Tiber River sediments (Rome. Italy): Implications on the post-glacial sea-level rise and Holocene climate. *Global and Planetary Change* **107**, 157-176 (2013).

43] Salomon, F. Géoarchéologie du delta du Tibre: évolution géomorphologique holocène et contraintes hydrosédimentaires dans le système Ostie-Portus (Italie). Thèse de doctorat en géographie. option géoarchéologie. *Physio-Géo. Géographie physique et environnement* **8,** 1-6 (2014).

44] Goiran, J. P., Tronchère, H., Collalelli, U., Salomon, F., Djerbi, H. Découverte d'un niveau marin biologique sur les quais de Portus: le port antique de Rome. *Méditerranée,* **112**, 59-67 (2009).

45] De Muro, S., Orrù, P. Il contributo delle Beach-Rock nello studio della risalita del mare olocenico. Le Beach-Rock post-glaciali della Sardegna nord-orientale. *Il Quaternario* **111**, 19-39, (1998).

46] Melis, R. T., et al. 8000 years of coastal changes on a western Mediterranean island: A multiproxy approach from the Posada plain of Sardinia. *Marine Geology* **403**, 93-108. (2018).

47] Melis, R. T., Depalmas, A., Di Rita, F., Montis, F., Vacchi, M. Mid to late Holocene environmental changes along the coast of western Sardinia (Mediterranean Sea). *Global and Planetary Change* **155**, 29-41 (2017).

48] Di Rita, F., Melis, R.T. The cultural landscape near the ancient city of Tharros (central West Sardinia): vegetation changes and human impact. *Journal of Archaeological Science* **40(12)**, 4271-4282 (2013).

49] De Falco, G., et al. Early cementation and accommodation space dictate the evolution of an overstepping barrier system during the Holocene. *Marine Geology* **369**, 52-66 (2015).

50] Ruiz, J. M., Carmona, P., Gómez-Bellard, C., van Dommelen, P. Geomorfología y cambio ambiental en el entorno de los yacimientos púnicos de la llanura de Terralba (Golfo de Oristano, isla de Cerdeña, Italia). *Boletín Geológico y Minero* **129**, 331-52 (2018).

51] Orrù, P., Solinas, E., Puliga, G., Deiana, G. Palaeo-shorelines of the historic period. Sant'Antioco Island. south-western Sardinia (Italy). *Quaternary International* **232(1)**, 71-81 (2011).

52] Orrù, P. E., et al. Sea level changes and geoarchaeology between the bay of Capo Malfatano and Piscinnì Bay (SW Sardinia) in the last 4 kys. *Quaternary International* **336**, 180-189 (2014).

53] Orrù, P. E., Antonioli, F., Lambeck, K., Verrubbi. V. Holocene sea-level change in the Cagliari coastal plain (southern Sardinia. Italy). *Quaternaria Nova* **8**, 193-212 (2004).

54] Barra D., et al. The Versilian transgression in the Volturno river plain (Campania. Southern Italy): Palaeoenvironmental history and chronological data. *Il Quaternario* **9(2)**, 445-458 (1996).

55] Sacchi, M., et al.Late-Holocene to recent evolution of Lake Patria. South Italy: An example of a coastal lagoon within a Mediterranean delta system. *Global and Planetary Change* **117**, 9-27 (2014).

56] Barra, D., et al. Depositional history and palaeogeographic reconstruction of Sele coastal plain during Magna Grecia settlement of Hera Argiva (Southern Italy). *Geologica Romana* **35**, 151-166, (1999).

57] Amato, V., et al. Relative sea level changes and paleogeographical evolution of the southern Sele plain (Italy) during the Holocene. *Quaternary International* **288**, 112-128 (2013).

58] Barra, D., et al. New data on the evolution of the Sele River coastal plain (southern Italy) during the Holocene. *Il Quaternario* **11**, 287–299 (1998).

59] Antonioli, F., Chemello, R., Improta, S., Riggio, S. The Dendropoma (Mollusca Gastropoda. Vermetidae) intertidal reef formations and their paleoclimatological use. *Marine Geology* **161**, 155-170, (1999).

60] Basso, D., Bernasconi, M. P., Robba, E., Marozzo, S. Environmental evolution of the Marsala sound. Sicily. during the last 6000 years. *Journal of Coastal Research* **24(1)** , 177-197 (2008).

61] Spampinato, C. R., Costa, B., Di Stefano, A., Monaco, C., Scicchitano, G. 2011. The contribution of tectonics to relative sea-level change during the Holocene in coastal south-eastern Sicily: new data from boreholes. *Quaternary International* **232(1)**, 214-227 (2011).

62] Gerardi. F., et al. Geological record of tsunami inundations in Pantano Morghella (south-eastern Sicily) both from near and far-field sources. *Natural Hazards and Earth System Sciences* **12(4),** 1185-1200 (2012).

63] Marriner, N., Gambin, T., Djamali, M., Morhange, C., Spiteri, M. Geoarchaeology of the Burmarrad ria and early Holocene human impacts in western Malta. *Palaeogeography, Palaeoclimatology, Palaeoecology,* **339**, 52-65 (2012).

64] Furlani, S. et al. Submerged speleothem in Malta indicates tectonic stability throughout the Holocene. *The Holocene* **28(10)**, 1588-1597 (2018).

65] Zaîbi, C., et al. Évolution du trait de côte à l'Holocène supérieur dans la Sebkha El-Guettiate de Skhira (Golfe de Gabès. Tunisie) à travers sa faune d'ostracodes et de foraminifères. *Geobios* **44(1)**, 101-115 (2011).

66] Zaïbi, C., et al. Evolution of the sebkha Dreîaa (South-Eastern Tunisia, Gulf of Gabes) during the Late Holocene: response of ostracod assemblages. *Revue de micropaléontologie* **55(3)**, 83-97 (2012).

67] Morhange. C., & Pirazzoli. P. A. Mid-Holocene emergence of southern Tunisian coasts. *Marine Geology* **220(1)**. 205-213 (2005).

68] Strasser, A., Davaud, E., Jedoui, Y. Carbonate cements in Holocene beachrock: example from Bahiret et Biban. southeastern Tunisia. *Sedimentary Geology* **62(1)**. 89-100 (1989).

69] Lakhdar, R., Soussi, M., Ben Ismail, M. H., M'Rabet, A. A Mediterranean Holocene restricted coastal lagoon under arid climate: Case of the sedimentary record of Sabkha Boujmel (SE Tunisia). *Palaeogeography, Palaeoclimatology, Palaeoecology* **241(2)**, 177-191 (2006).

70] Serandrei-Barbero, R., Albani, A., Donnici, S., Rizzetto, F. Past and recent sedimentation rates in the Lagoon of Venice (Northern Italy). *Estuarine, Coastal and Shelf Science* **69(1)**, 255-269, (2006)

71] McClennen, C.E., & Housley. R.A. Late-Holocene channel meander migration and mudflat accumulation rates. lagoon of Venice, Italy. *Journal of Coastal Research* **224,** 930-945 (2006).

72] Zoppi, U., et al. Preliminary estimate of the reservoir age in the Lagoon of Venice. *Radiocarbon* **43(2A)**, 489-494 (2001).

73] Madricardo, F., & Donnici, S. Mapping past and recent landscape modifications in the Lagoon of Venice through geophysical surveys and historical maps. *Anthropocene* **6,** 86-96 (2014).

74] Tosi. L., Foglio geologico 128 Venezia e note illustrative. *Carta geologica d'Italia alla scala 1: 50.000* (2007).

75] Donnici, S., Serandrei-Barbero, R., Canali, G. Evidence of climatic changes in the Venetian Coastal Plain (Northern Italy) during the last 40,000 years. *Sedimentary Geology* **281**, 139-150 (2012).

76] Galassi, P., & Marocco, R. Relative sea level rise and subsidence in the Caorle Lagoon (Northern Adriatic Sea). Italy. during Holocene. *Il Quaternario* **12**, 249-256 (1999).

77] Fontana. A., et al. Lagoonal settlements and relative sea level during Bronze Age in Northern Adriatic: Geoarchaeological evidence and paleogeographic constraints. *Quaternary International* **439**, 17-36 (2017).

78] Fontana, A. & Zanferrari. A. Foglio geologico 86 San Vito al Tagliamento e note illustrative. *Carta geologica d'Italia alla scala 1: 50.000* (2006).

79] Bondesan, A., Meneghel, M., Rosselli, R., Vitturi. A. Geomorphological Map of the Province of Venice. scale 1: 50.000. LAC. Firenze (4 sheets) (2004).

80] Furlani. S., et al. Tectonic effects on Late Holocene sea level changes in the Gulf of Trieste (NE Adriatic Sea. Italy). *Quaternary International* **232(1)**, 144-157 (2011).

81] Covelli, S., Fontolan, G., Faganeli, J., Ogrinc, N. Anthropogenic markers in the Holocene stratigraphic sequence of the Gulf of Trieste (northern Adriatic Sea). *Marine Geology* **230(1-2)**, 29-51 (2006).

82] Melis, R., et al. Sea level and paleoenvironment during roman times inferred from coastal archaeological sites in Trieste (northern Italy). *Alpine and Mediterranean Quaternary* **25(1)**, 41-55 (2012).

83] Ogorelec, B., Faganeli, J., Mišič, M., Čermelj, B. Reconstruction of paleoenvironment in the Bay of Koper (Gulf of Trieste, northern Adriatic). *Annales* **11(187)**, e200 (1997).

84] Faivre, S., et al Relative sea level change in western Istria (Croatia) during the last millennium. *Quaternary international* **232(1-2)**, 132-143 (2011).

85] Cibin U. Stefani. M. Foglio geologico 187 Codigoro e note illustrative. *Carta geologica d'Italia alla scala 1: 50.000*. (2009).

86] Correggiari, A., Roveri, M., Trincardi, F. Late Pleistocene andHolocene evolution of the north Adriatic Sea. *Il Quaternario* 9, 697-704 (1996).

87] Sarti, G., Centineo, M.C., Calabrese, L. Foglio geologico 205 Comacchio e note illustrative. *Carta geologica d'Italia alla scala 1: 50.000*. (2009).

88] Marriner, N., et al. Post-Roman sea-level changes on Pag Island (Adriatic Sea): Dating Croatia's "enigmatic" coastal notch? *Geomorphology* **221**, 83-94 (2014).

89] Faivre, S., Bakran-Petricioli, T., Horvatinčić, N., Sironić, A. Distinct phases of relative sea level changes in the central Adriatic during the last 1500years—influence of climatic variations?. *Palaeogeography. Palaeoclimatology. Palaeoecology* **369**, 163-174 (2013).

90] Caldara, M., Caroli, I., Simone, O. Holocene evolution and sea-level changes in the Battaglia basin area (eastern Gargano coast. Apulia. Italy). *Quaternary International* **183(1)**, 102-114 (2008).

91] Caldara, M., Simone, O. Coastal changes in the eastern Tavoliere Plain (Apulia. Italy) during the Late Holocene: Natural or anthropic? *Quaternary Science Reviews* **24(18)**, 2137-2145 (2005).

92] Di Rita, F., Simone, O., Caldara, M., Gehrels, W. R., Magri, D. Holocene environmental changes in the coastal Tavoliere Plain (Apulia, southern Italy): a multiproxy approach. *Palaeogeography, Palaeoclimatology, Palaeoecology* **310(3-4)**, 139-151 (2011).

93] Primavera, M., Simone, O., Fiorentino, G., Caldara, M. The palaeoenvironmental study of the Alimini Piccolo lake enables a reconstruction of Holocene sea-level changes in southeast Italy. *The Holocene* **21(4)**, 553-563 (2011).
